# Supplementary material for: Potentiating cancer immunotherapies with modular albumin-hitchhiking nanobody–STING agonist conjugates
Source: Nat Biomed Eng. 2025 Jun 11;9(10):1719–39. doi: 10.1038/s41551-025-01400-0 (PMC12532571; doi:10.1038/s41551-025-01400-0)
Supplement: Supplementary file 1 — Supplementary Figs. 1–42 and Tables 1–5. [file 41551_2025_1400_MOESM1_ESM.pdf]

nature biomedical engineering

Supplementary information

https://doi.org/10.1038/s41551-025-01400-0

Potentiating cancer immunotherapies with modular albumin-hitchhiking nanobody–STING agonist conjugates

In the format provided by the authors and unedited

Contents:

Supplementary Figure 1: Isothermal calorimetry (ITC) plots.
Supplementary Figure 2: Synthesis of DBCO-PEG_{11}-diABZI.
Supplementary Figure 3: 1H NMR 500 MHz of DBCO-PEG_{11}-diABZI in DMSO.
Supplementary Figure 4: 13C NMR 151 MHz of DBCO-PEG_{11}-diABZI in DMSO.
Supplementary Figure 5: ESI-MS characterization of synthesis products.
Supplementary Figure 6: Tritosome degradation assay.
Supplementary Figure 7: Synthesis of Amine-PEG_3-Triazole-PEG_{11}-diABZI.
Supplementary Figure 8: 1H NMR 500 MHz of Amine-PEG_3-Triazole-PEG_{11}-diABZI in DMSO.
Supplementary Figure 9: MALDI-TOF MS of Amine-PEG_3-Triazole-PEG_{11}-diABZI.
Supplementary Figure 10: *In vitro* dose-response curves for THP1-Dual reporter cells.
Supplementary Figure 11: Expanded fluorescent microscopy images from tumor sections at a 24 h time point.
Supplementary Figure 12: Western blot and fluorescent microscopy images of SPARC in tumor cell lines.
Supplementary Figure 13: Flow cytometric analysis for nAlb-Cy5 cellular uptake in EMT6 tumors and spleens.
Supplementary Figure 14: Total Cy5 positive tumor cells of the live population.
Supplementary Figure 15: Free diABZI (15 µg) and nAlb-diABZI (5, 0.5, 0.05 µg) I.V. injections in B16.F10 bearing mice.
Supplementary Figure 16: Mouse weight loss curves during treatment with diABZI and diABZI conjugated proteins.
Supplementary Figure 17: NanoString panel for evaluating STING activation markers within EMT6 tumor bearing Balb/C mice 24 h after three doses.
Supplementary Figure 18: Heat map indicating fold changes differences of indicated cell population spleens from Balb/c mice bearing EMT6 tumors**.**
Supplementary Figure 19: MDSC inhibitor treatment using SX-682 compared to regular chow.
Supplementary Figure 20: EMT6 tumor inoculation and treatment schedule with anti-GR-1 MDSC depletion.
Supplementary Figure 21: Anti-VHH antibody titer for nAlb response.
Supplementary Figure 22: Pharmacokinetics of nAlb-Cy5 and pre-treated nAlb-Cy5.
Supplementary Figure 23: *In vitro* qPCR analysis of genes associated with STING activation in bone marrow derived dendritic cells.

Supplementary Figure 24: *In vitro* dose-response curves and toxicity profiles.

Supplementary Figure 25: Tumor growth curves for treated EMT6 W.T. and EMT6 PD-L1 K.O. tumors.

Supplementary Figure 26: EMT6 serum cytokine/chemokine concentrations 4 h post-injection.

Supplementary Figure 27: Gene expression analysis in nAlb-diABZI + ICB vs. PBS.

Supplementary Figure 28: Tumor flow cytometry analysis of EMT6 bearing mice for MDSC depletion using anti-GR-1 antibodies.

Supplementary Figure 29: Spleen flow cytometry heat maps 48 h after administration of one therapy dose.

Supplementary Figure 30: tSNE plots of live cells from female EMT6 tumor-bearing Balb/c mice.

Supplementary Figure 31: Heat maps summarizing the fold change in the frequency of NK cells, $CD8^+$ T cells, and $CD4^+$ T cells.

Supplementary Figure 32: Analysis of the frequency of tumor cells after two doses of AP-diABZI or PBS.

Supplementary Figure 33: B16.F10 tumor inoculation and treatment schedule.

Supplementary Figure 34: Representative images of H&E-stained lung sections.

Supplementary Figure 35: Representative flow cytometry dot plots and gating strategy showing the relative uptake of Cy5 in each cell population in the tumor.

Supplementary Figure 36: Representative flow cytometry dot plots and gating strategy showing the cell populations present in the tumor.

Supplementary Figure 37: Representative flow cytometry dot plots and gating strategy showing the relative uptake of Cy5 in each cell population in the spleen.

Supplementary Figure 38: Representative flow cytometry analysis for cell population mapping and immunophenotype determination within tumors and spleens from EMT6-bearing mice.

Supplementary Figure 39: Representative flow cytometry analysis denoting activation, proliferation, antigen presentation, and checkpoint markers for CD45+ cells.

Supplementary Figure 40: Representative flow cytometry analysis to map cell populations for tumors and spleens from EMT6-bearing mice (Balb/c).

Supplementary Figure 41: Representative flow cytometric gating strategy for spleen cells from B16.F10 bearing mice (C57BL/6).

Supplementary Figure 42: Representative flow cytometric analysis of CD4, CD8 T cells, and SIINFEKL/H-2kB stained tetramer CD8 T cells.

Supplementary Table 1: Protein sequences generated.

Supplementary Table 2: Antibodies for western blots.
Supplementary Table 3: Cy5 uptake flow cytometry panel (Balb/c).
Supplementary Table 4: Cell population and immunophenotyping flow cytometry panel (Balb/c).
Supplementary Table 5: Antibodies for immune cell memory in B16.F10-OVA tumor model.

Supplementary Figures:

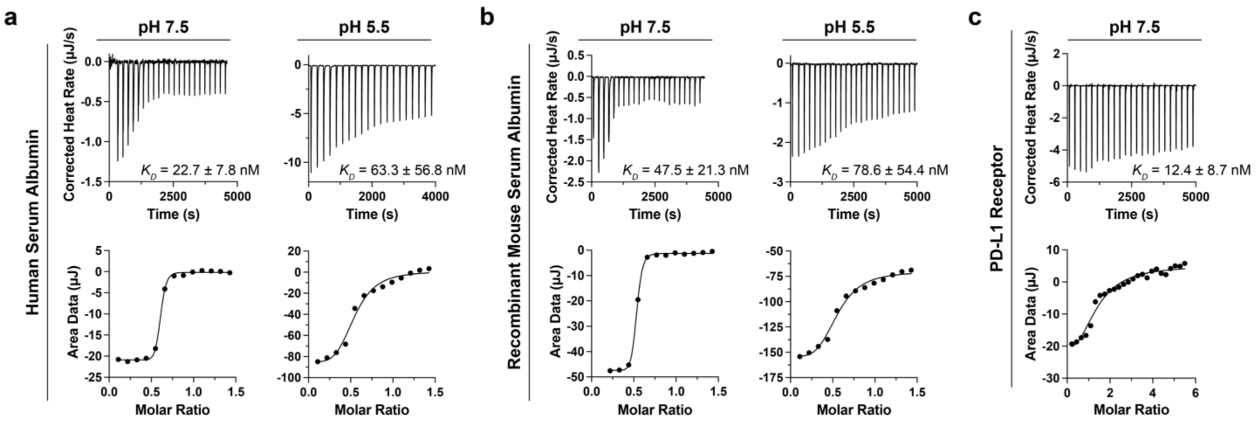

Supplementary Figure 1: Isothermal calorimetry (ITC) for nAlb at pH 7.5 and 5.5 in **(a)** human serum albumin and **(b)** recombinant mouse serum albumin. **(c)** nPD-L1 at pH 7.5 with PD-L1 receptor.

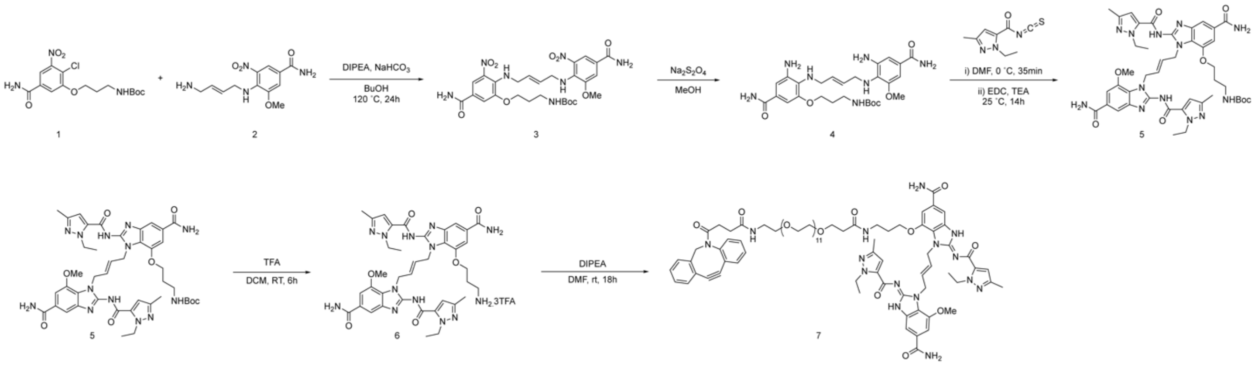

Supplementary Figure 2: Synthesis of DBCO-PEG$_{11}$-diABZI (**7**).

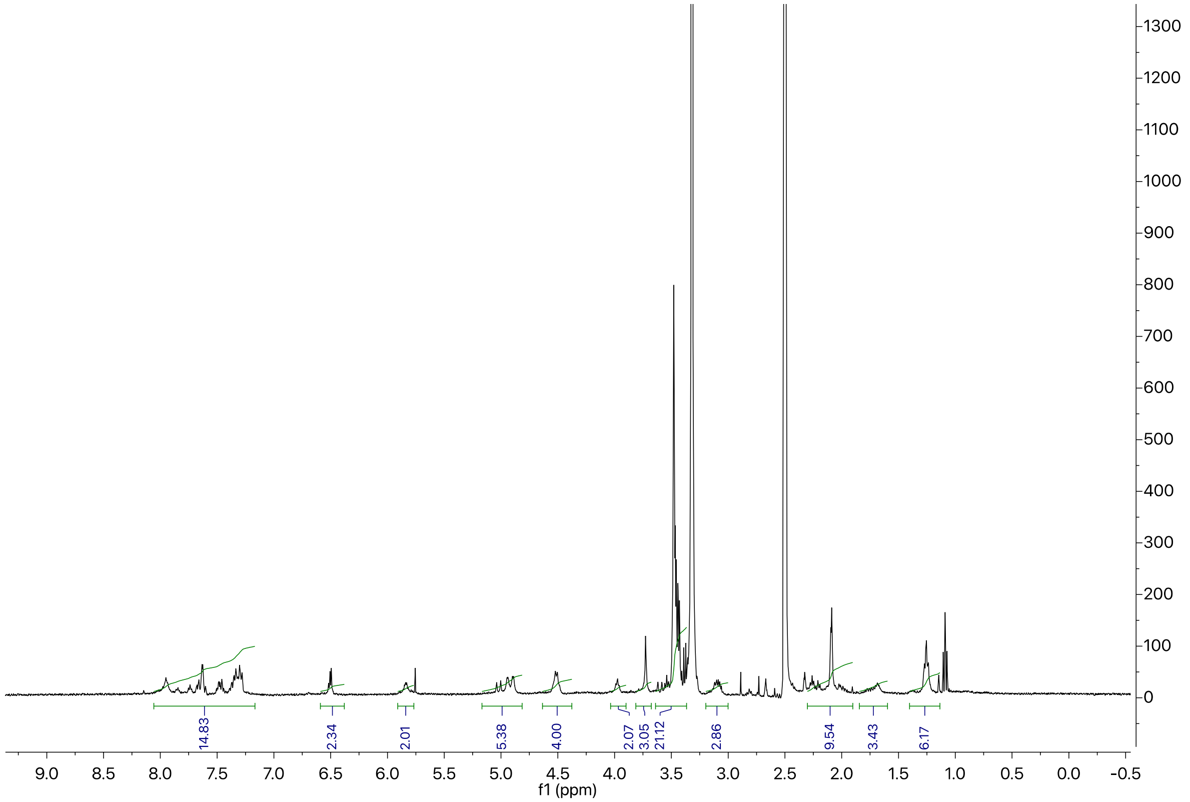

Supplementary Figure 3: 1H NMR 500 MHz of DBCO-PEG$_{11}$-diABZI (**7**) in DMSO.

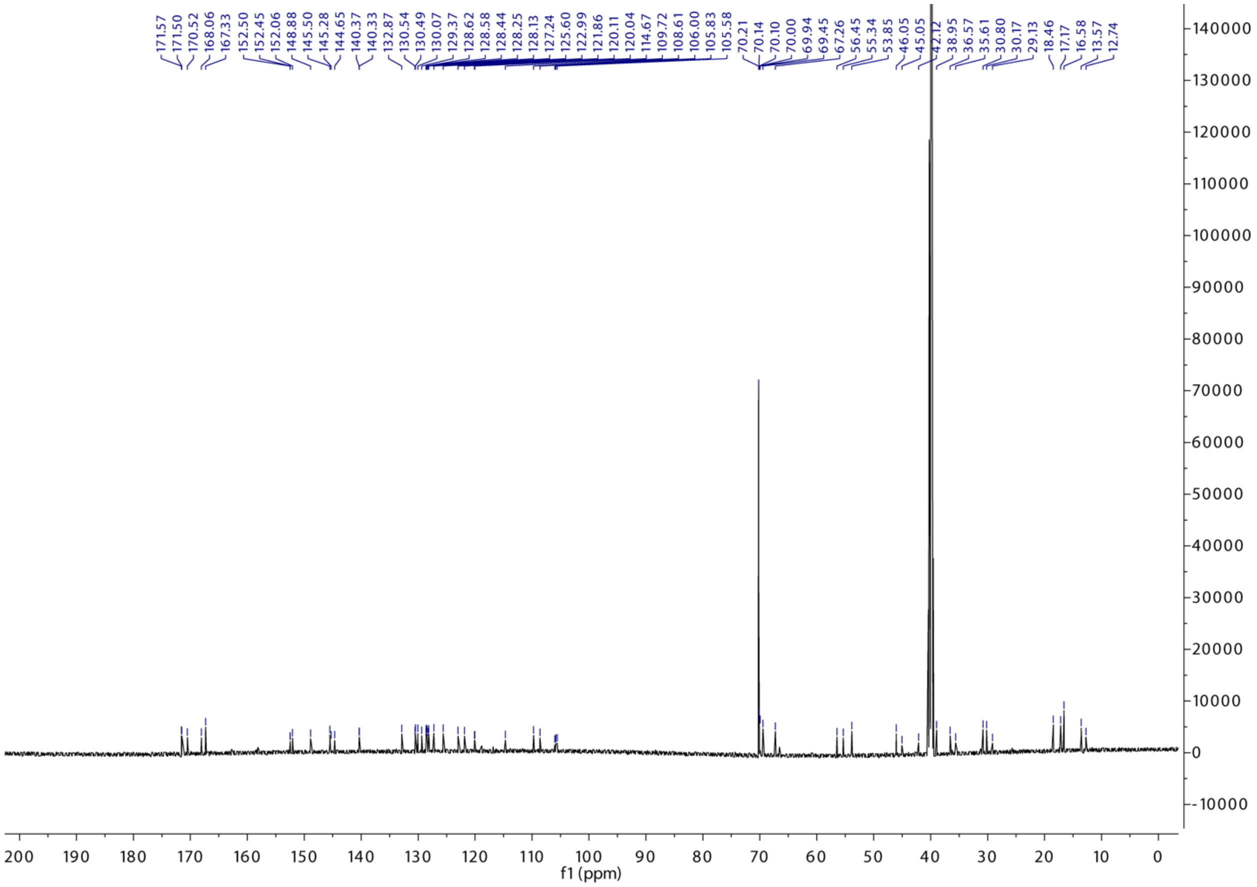

Supplementary Figure 4: 13C NMR 151 MHz of DBCO-PEG$_{11}$-diABZI (**7**) in DMSO.

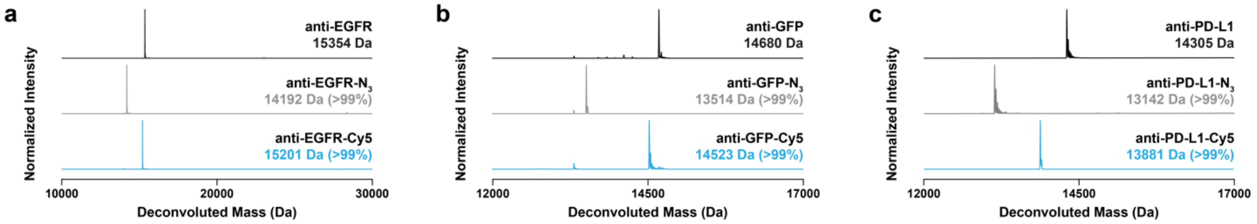

Supplementary Figure 5: ESI-MS characterization of synthesis products for **(a)** nEGFR, **(b)** nGFP, and **(c)** nPD-L1.

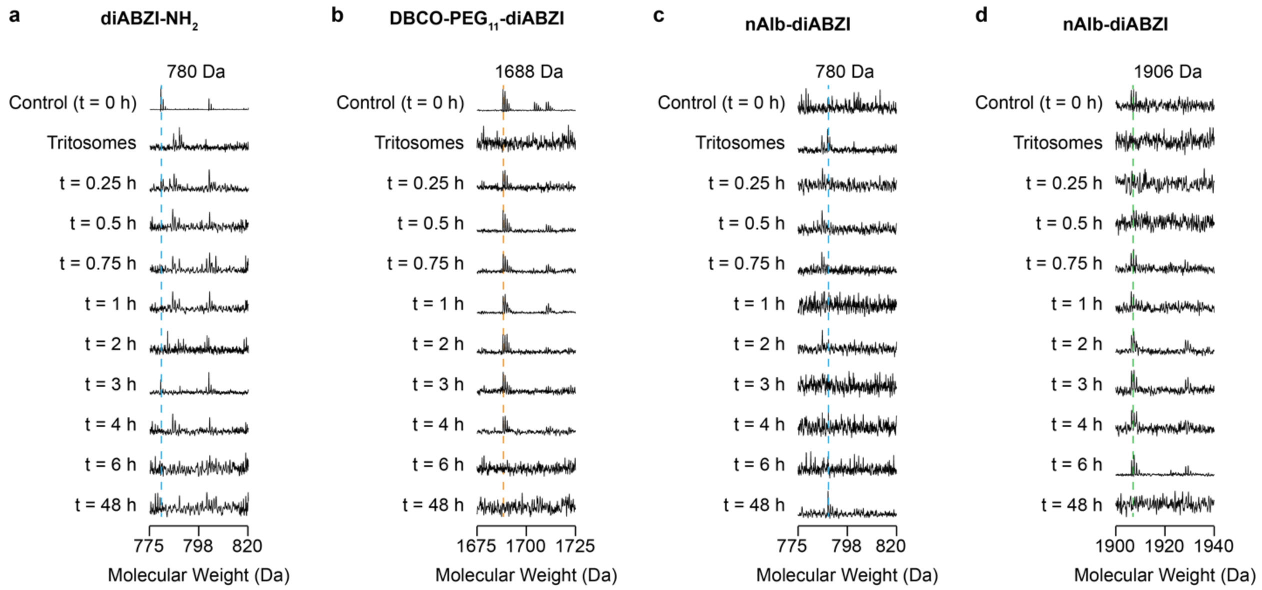

Supplementary Figure 6: Tritosome degradation assay for **(a)** diABZI-amine, **(b)** DBCO-PEG_{11}-diABZI, and **(c-d)** nAlb-amine with highlighted molecular weight ranges at 780 Da (diABZI-amine construct, blue dashed line), 1688 Da (DBCO-PEG_{11}-diABZI construct, orange dashed line), and 1906 Da (Amine-PEG_3-Triazole-PEG_{11}-diABZI).

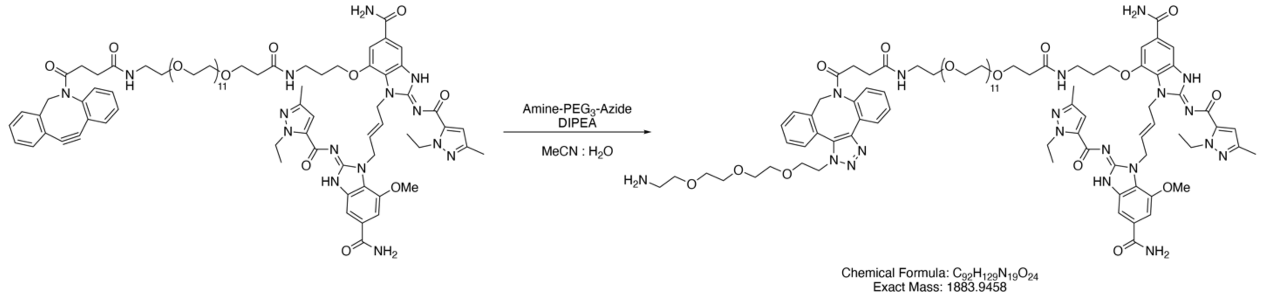

Supplementary Figure 7: Synthesis of Amine-PEG$_3$-Triazole-PEG$_{11}$-diABZI.

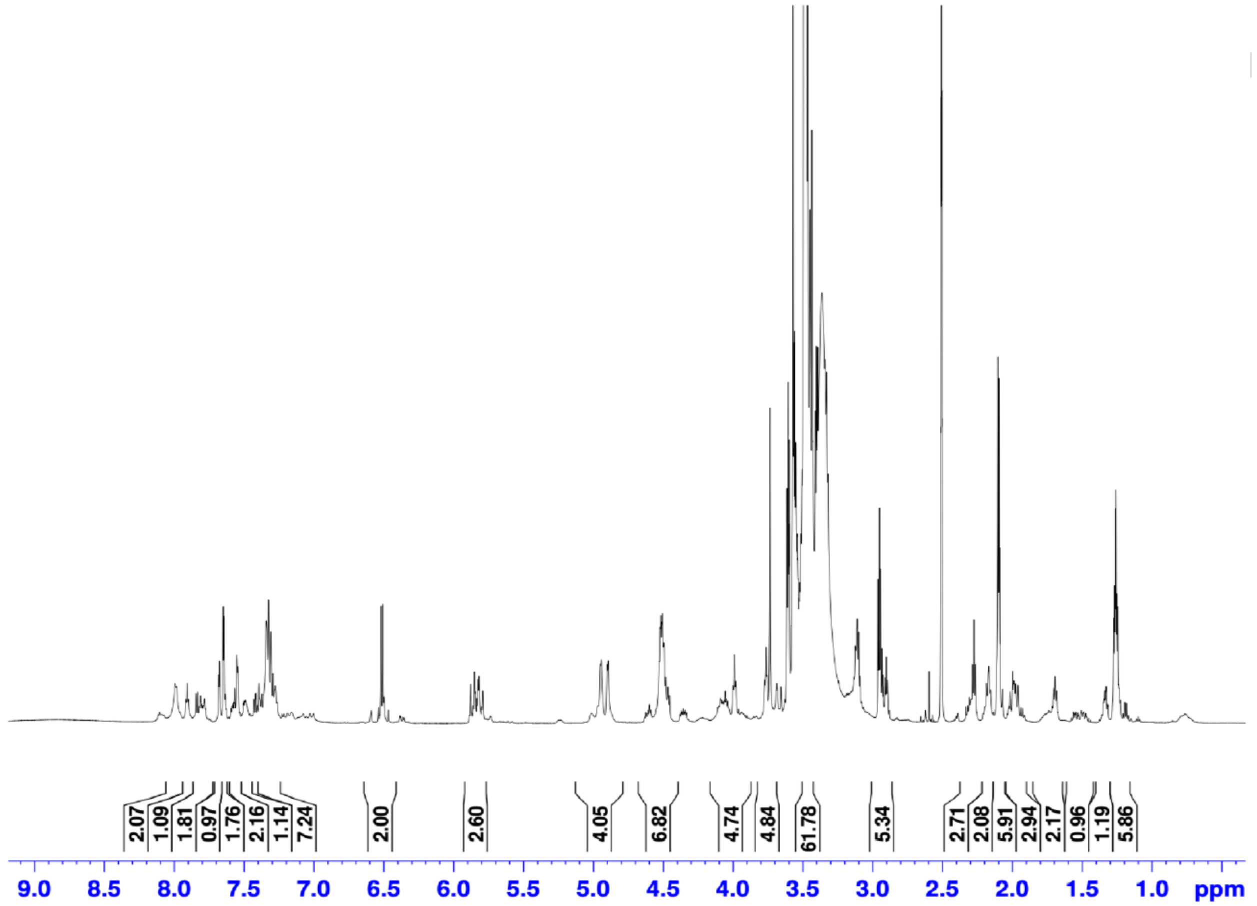

Supplementary Figure 8: 1H NMR 500 MHz of Amine-PEG$_3$-Triazole-PEG$_{11}$-diABZI in DMSO.

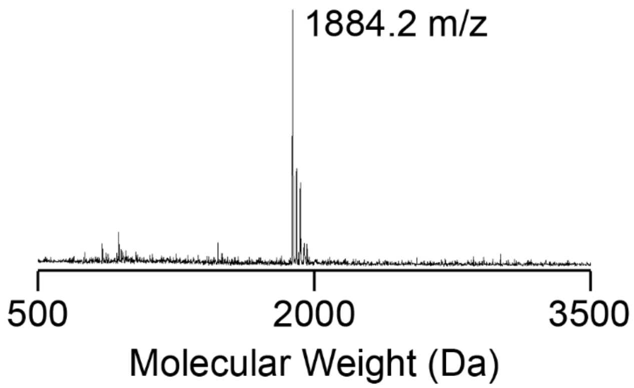

Supplementary Figure 9: MALDI-TOF MS of Amine-PEG$_3$-Triazole-PEG$_{11}$-diABZI.

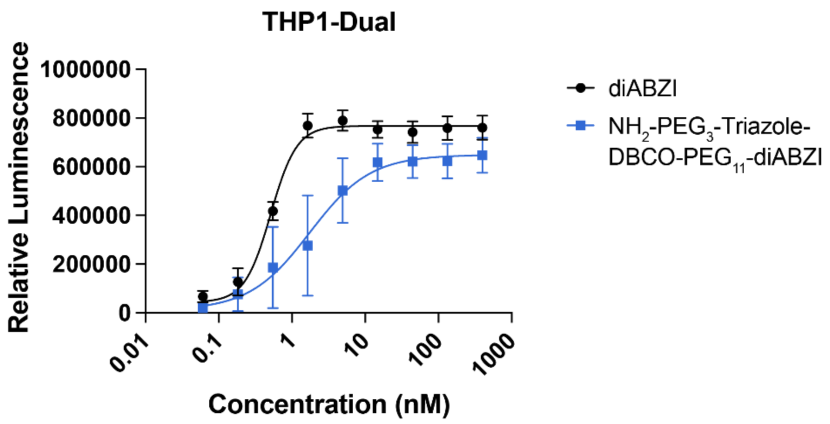

Supplementary Figure 10: *In vitro* dose-response curves for THP1-Dual reporter cells comparing diABZI to Amine-PEG_3-Triazole-PEG_{11}-diABZI (n=3). Replicates are biological, and data are shown as mean ± SEM.

.

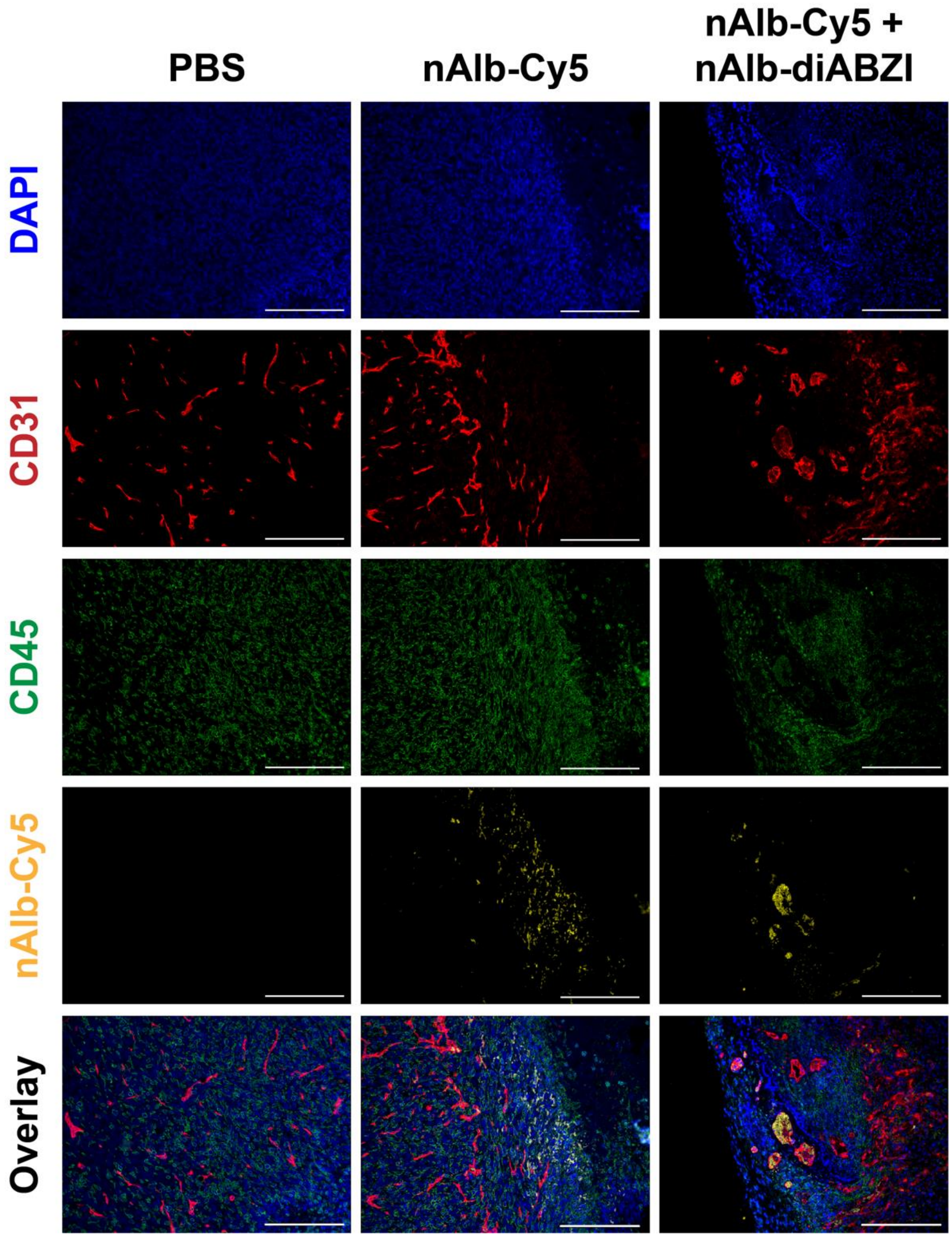

Supplementary Figure 11: Expanded fluorescent microscopy images at a 24 h time point from tumor sections following administration of nAlb-Cy5 (yellow) alone or in combination with nAlb-diABZI, and stained for DAPI (blue), CD45 (green), and CD31 (red) (scale bar: 200 μm).

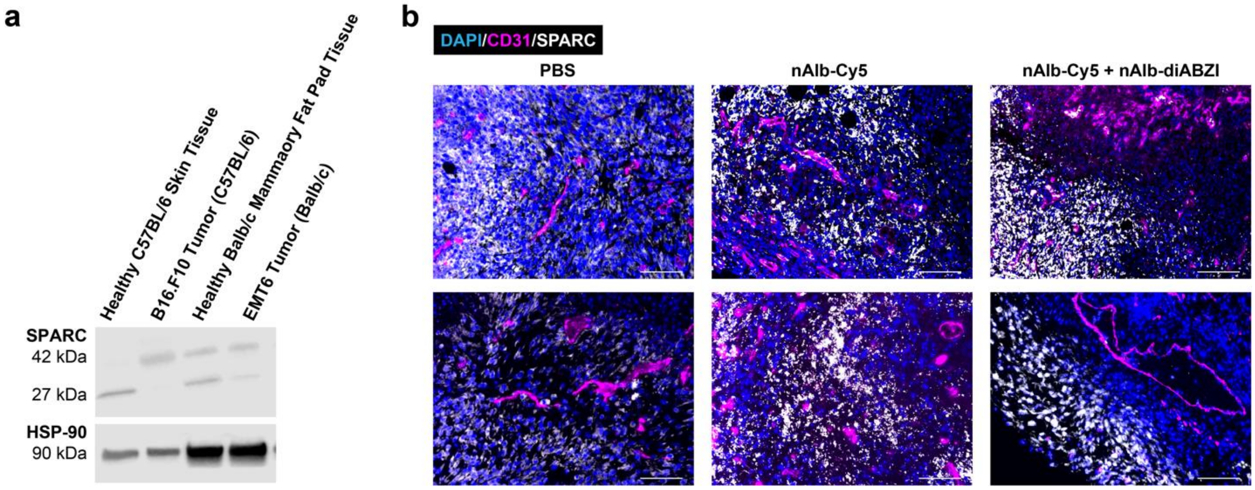

Supplementary Figure 12: (a) Western blot analysis of SPARC at ~42 kDa and a proteolytically cleaved fragment at ~27kDa and HSP-90 (90 kDa) proteins in B16.F10 and EMT6 tumor tissue (see Source Data for uncropped western blot images). **(b)** Representative fluorescent microscopy images of EMT6 breast tumor sections stained with DAPI (blue), anti-CD31 antibody (magenta), and anti-SPARC antibody (white) 24 h following administration of PBS, nAlb-Cy5, or nAlb-diABZI in combination with nAlb-diABZI (scale bar: 100 µm).

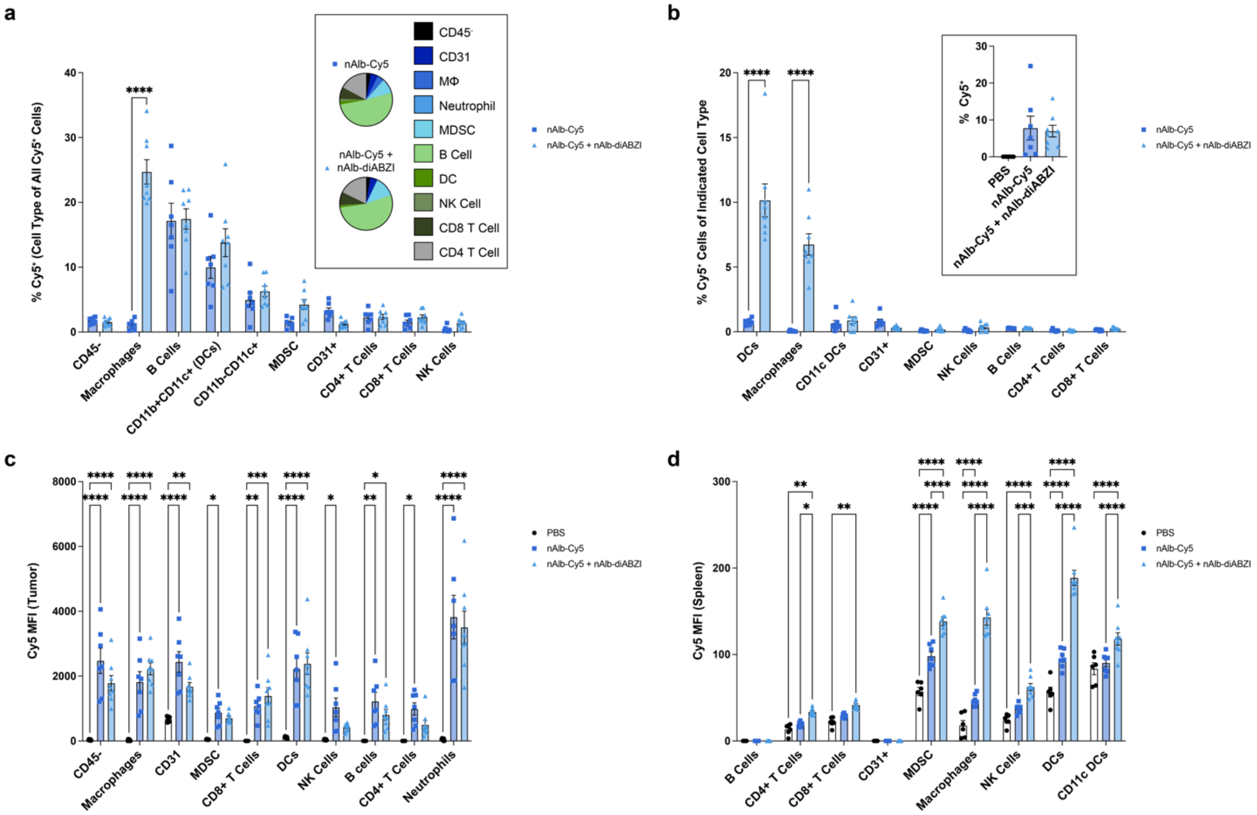

Supplementary Figure 13: Flow cytometric analysis for nAlb-Cy5 cellular uptake in EMT6 spleens and tumors. **(a-b)** Populations percentages of **(a)** total Cy5 positive cells in spleen and **(b)** of cells from indicated cell types with comparisons of nAlb-Cy5 with and without treatment of nAlb-diABZI, inset: percentage of $Cy5^+$ cells in spleen. **(c-d)** Median fluorescent intensities (MFI) for each cell population in **(c)** tumors and **(d)** spleens. DC: dendritic cell; Mφ: macrophage; MDSC: myeloid derived suppressor cell; NK: natural killer cell (n=6 for PBS; n=7 for nAlb-Cy5; n=8 for nAlb-Cy5 + nAlb-diABZI). *P* values determined by ordinary two-way ANOVA with (a-b) with post-hoc Bonferroni's correction for multiple comparisons and (c-d) with post-hoc Tukey's correction for multiple comparisons; *$P \leq 0.05$, **$P \leq 0.01$, ***$P \leq 0.001$, and ****$P < 0.0001$. Replicates are biological, and data are shown as mean ± SEM.

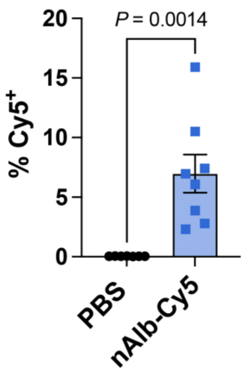

Supplementary Figure 14: Frequency of Cy5-positive live cells in EMT6 breast tumors as determined by flow cytometry (n=7 for PBS; n=9 for nAlb-Cy5). *P* value determined by two-sided Student’s t-test. Replicates are biological, and data are shown as mean ± SEM.

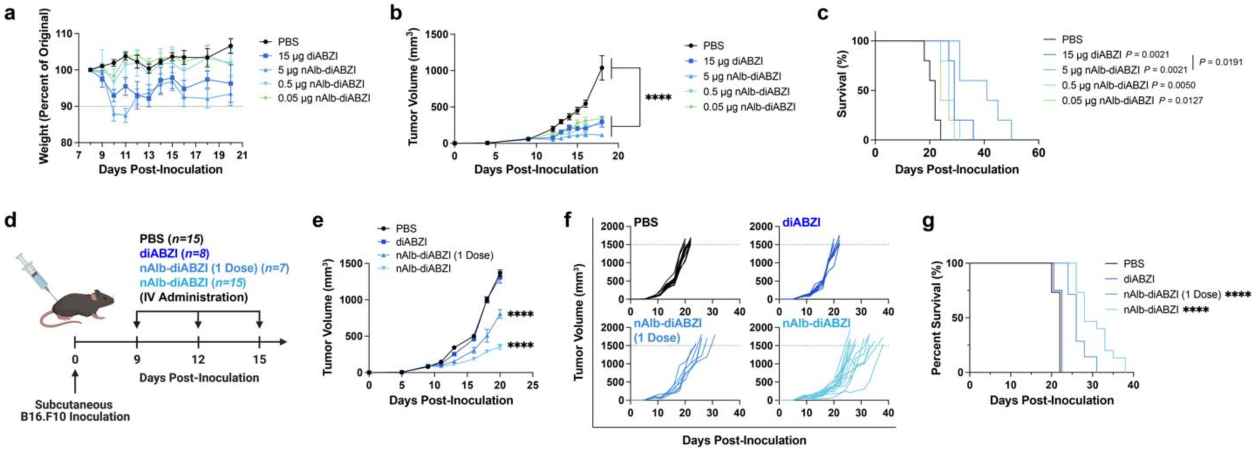

Supplementary Figure 15: Free diABZI (15 µg) and nAlb-diABZI (5, 0.5, 0.05 µg) were injected I.V. **(a)** Mouse weights, **(b)** Tumor growth curves, and **(c)** Kaplan-Meier survival plots for mice with B16.F10 tumors treated as indicated (n=5). **(d)** Schematic of B16.F10 tumor inoculation and treatment schedule. **(e)** Tumor growth curves, **(f)** spider plots of individual tumor growth curves, and **(g)** Kaplan-Meier survival plots for mice with B16.F10 tumors treated as indicated (n=15 for PBS and 3-dose nAlb-diABZI; n=8 for diABZI; n=7 for single-dose nAlb-diABZI). (b,e) Data represented with error in SEM with *P* value determined by two-way ANOVA with post-hoc Tukey's correction for multiple comparisons; *****P*<0.0001 on day 20 for all groups compared to PBS. (c,g) Endpoint criteria of 1500 mm^3 tumor volume with *P* value determined by log-rank test compared to PBS group or as indicated. Replicates are biological, and data are shown as mean ± SEM.

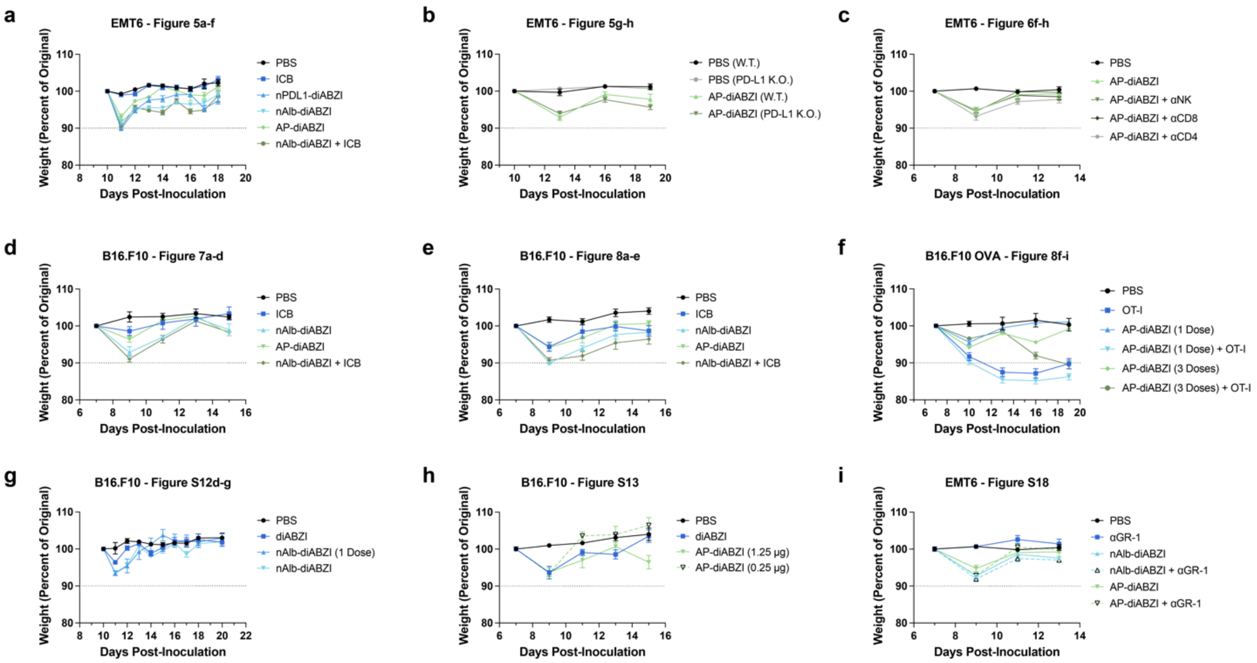

Supplementary Figure 16: Mouse weight loss curves during treatment course with diABZI and diABZI conjugated proteins from indicated studies as represented by subfigure titles; number of mice per group (n) are indicated in caption of figure noted in the subfigure title. Replicates are biological, and data are shown as mean ± SEM.

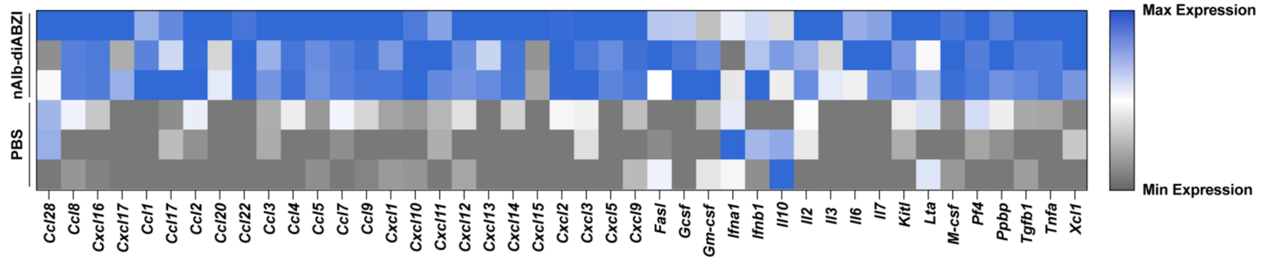

Supplementary Figure 17: NanoString panel for evaluating STING activation markers within EMT6 tumor bearing Balb/C mice 24 h after three doses. Groups used include nAlb-diABZI (1.25 μg) and PBS (n=3). Replicates are biological.

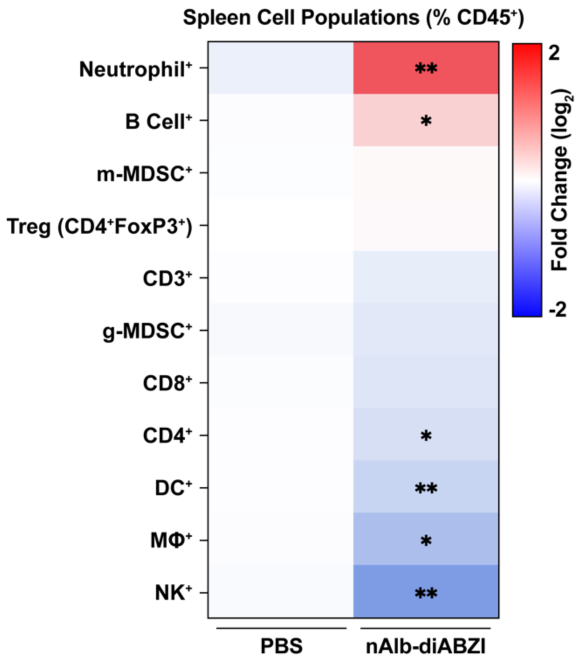

Supplementary Figure 18: Heat map indicating fold changes differences of indicated cell population spleens from Balb/c mice bearing EMT6 tumors treated as indicated. **P*≤0.05, ***P*≤0.01, ****P*≤0.001, and *****P*<0.0001 indicate a statistically significant difference between PBS and nAlb-diABZI treated groups as determined by two-tailed Student's t-test (n=6). Replicates are biological.

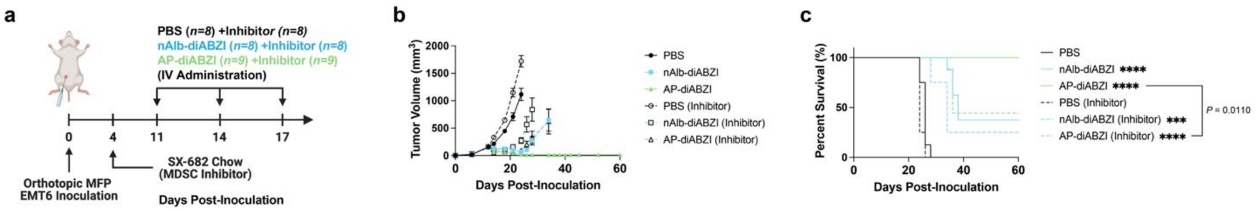

Supplementary Figure 19: (a) Schematic of EMT6 tumor inoculation and treatment schedule with nAlb-diABZI, AP-diABZI, or PBS (vehicle) combined with chow containing SX-682 (CXCR1/2 inhibitor) or regular chow. nAlb-diABZI and AP-diABZI are administered I.V and SX-682 is delivered orally in chow. **(b)** Tumor growth curves and **(c)** Kaplan-Meier survival plots (n=8 for PBS and nAlb-diABZI ± SX-682 and n=9 for AP-diABZI ± SX-682). (c) Endpoint criteria of 1500 mm^3 tumor volume with *P* value determined by log-rank test compared to PBS group or between AP-diABZI and AP-diABZI + SX-682 as indicated. Replicates are biological, and data are shown as mean ± SEM.

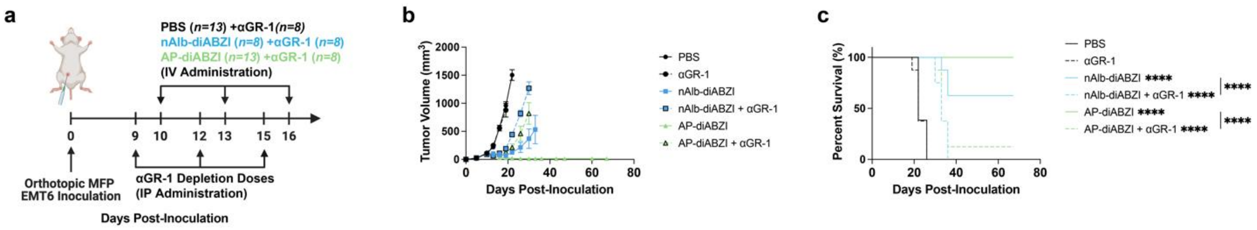

Supplementary Figure 20: (a) Schematic of EMT6 tumor inoculation and treatment schedule with nAlb-diABZI, AP-diABZI, or PBS (vehicle) combined with anti-GR-1 MDSC depletion. **(b)** Tumor growth curves and **(c)** Kaplan-Meier survival plots (n=13 for PBS and AP-diABZI; n=8 for all other groups). nAlb-diABZI and AP-diABZI are administered I.V and anti-GR-1 is administered I.P. (c) Endpoint criteria of 1500 mm^3 tumor volume with *P* value determined by log-rank test compared to PBS group or between groups as indicated. Replicates are biological, and data are shown as mean ± SEM.

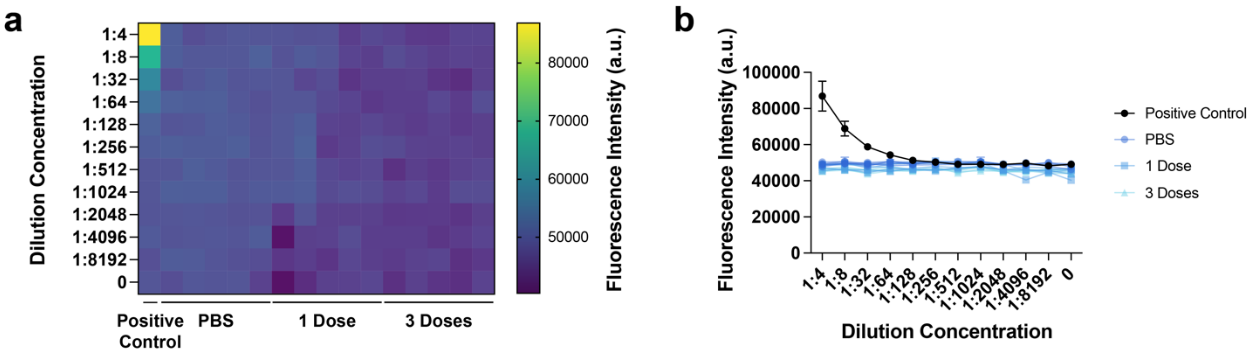

Supplementary Figure 21: Evaluation of anti-VHH antibody response elicited by I.V. administration of nAlb-diABZI. ELISA data summarized in **(a)** heat map and **(b)** average fluorescence as a function of serum dilution after treatment with one or three doses of nAlb-diABZI (n=5). Replicates are biological, and data are shown as mean ± SEM.

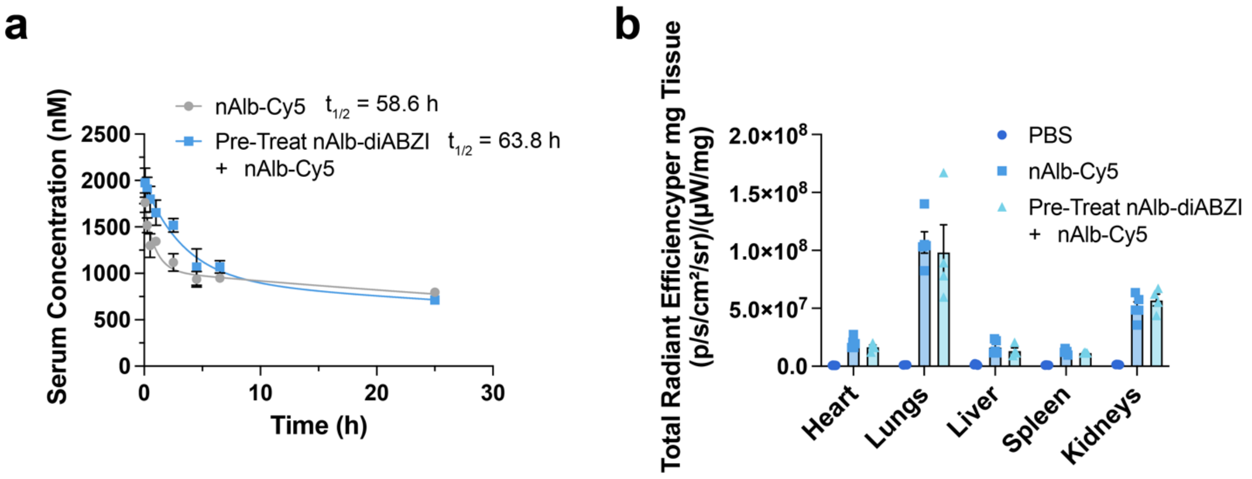

Supplementary Figure 22: (a) Pharmacokinetics of nAlb-Cy5 in untreated mice (n=5) or mice pre-treated with nAlb-diABZI (n=4) three times, spaced three days apart, with elimination phase half-life indicated in legend. **(b)** Quantification of radiant efficiencies 24 h following intravenous administration of PBS (n=5), nAlb-Cy5 (n=5), or nAlb-Cy5 after 3 doses of nAlb-diABZI (n=4). Replicates are biological, and data are shown as mean ± SEM.

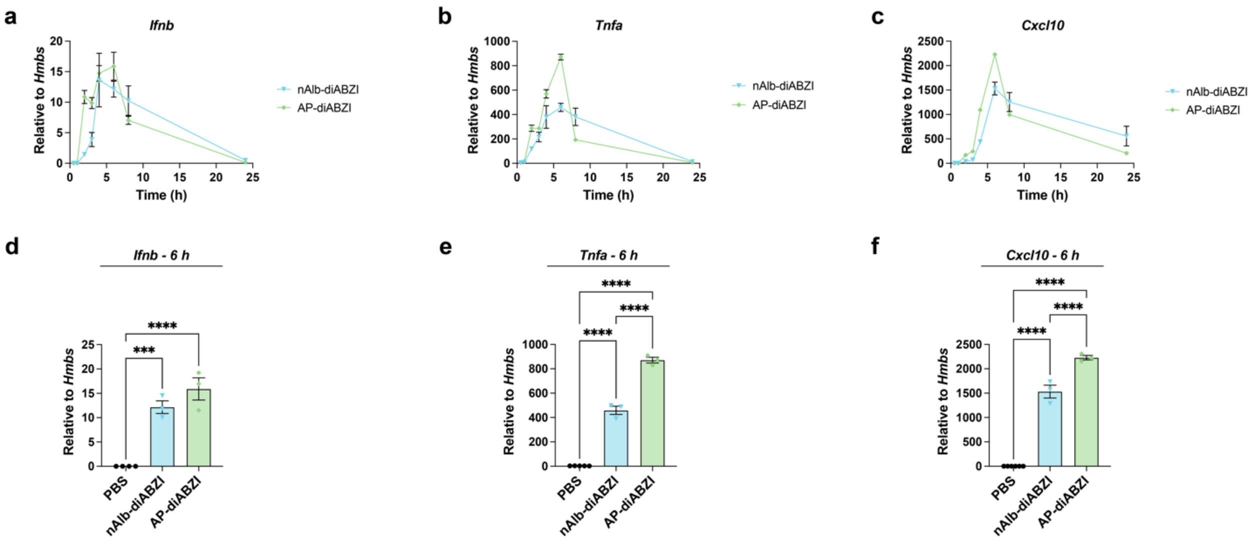

Supplementary Figure 23: *In vitro* qPCR analysis of genes associated with STING activation (*Ifnb*, *Tnfa*, and *Cxcl10*) in bone marrow derived dendritic cells (BMDCs) as analyzed (**a-c**) over 24 h (n=3) or (**d-f**) at 6 h post-treatment with nanobody-diABZI conjugates at 0.25 µM (n=4 for PBS; n=3 for other groups)). *P* values were determined by one-way ANOVA with post-hoc Tukey's correction for multiple comparisons; ***$P \leq 0.001$ and ****$P < 0.0001$. Replicates are biological, and data are shown as mean ± SEM.

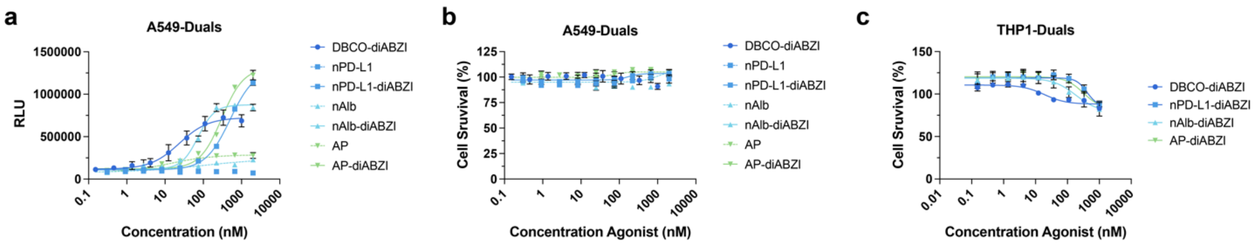

Supplementary Figure 24: (a) Dose-response curves for indicated nanobodies and nanobody-diABZI conjugates in A549-Dual IFN-I reporter cell lines (n=3). **(b,c)** Evaluation of cell viability following incubation with indicated nanobodies or nanobody-diABZI conjugates in **(b)** A549-Dual reporter cells and **(c)** THP1-Dual reporter cells measured by Cell-Titer Glo assay (n=3). Replicates are biological, and data are shown as mean ± SEM.

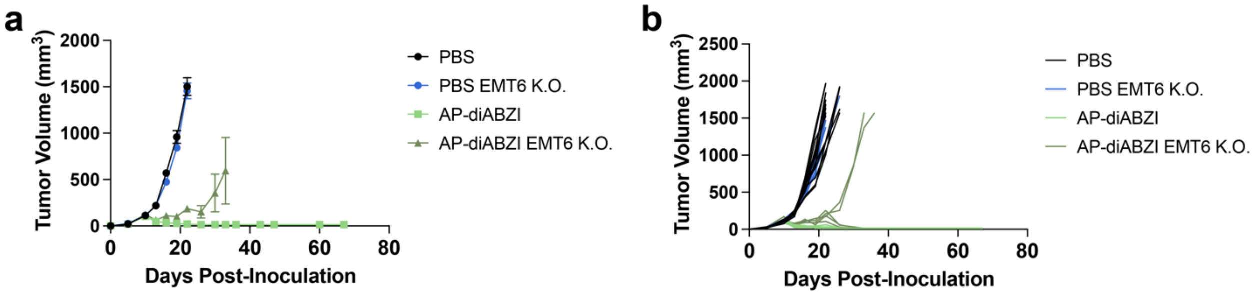

Supplementary Figure 25: (a) Tumor growth curves and **(b)** spider plots of individual tumor growth curves for mice with EMT6 W.T. and EMT6 PD-L1 K.O. tumors corresponding to study presented in **Fig. 5g-h**; n=13 for PBS and AP-diABZI and n=5 for both EMT6 K.O. groups. Replicates are biological, and data are shown as mean ± SEM.

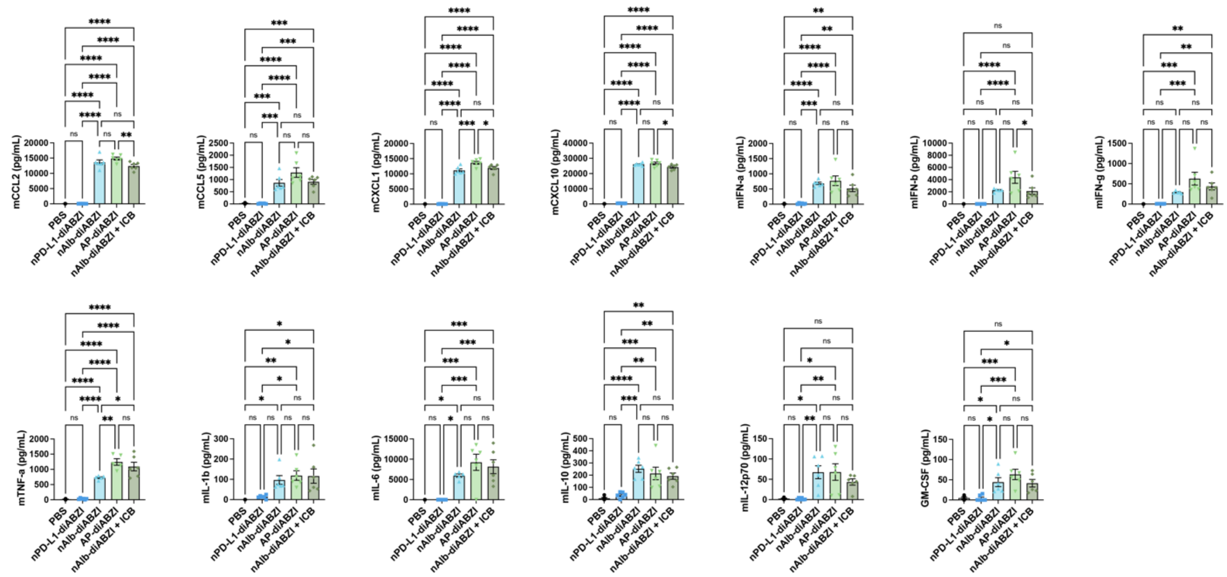

Supplementary Figure 26: Serum cytokine/chemokine concentrations 4 h post-injection with indicated nanobody-diABZI conjugate in Balb/c mice with EMT6 tumors (n=6). *P* values were determined by one-way ANOVA with post-hoc Tukey's correction for multiple comparisons; **P*≤0.05, ***P*≤0.01, ****P*≤0.001, and *****P*<0.0001. Replicates are biological, and data are shown as mean ± SEM.

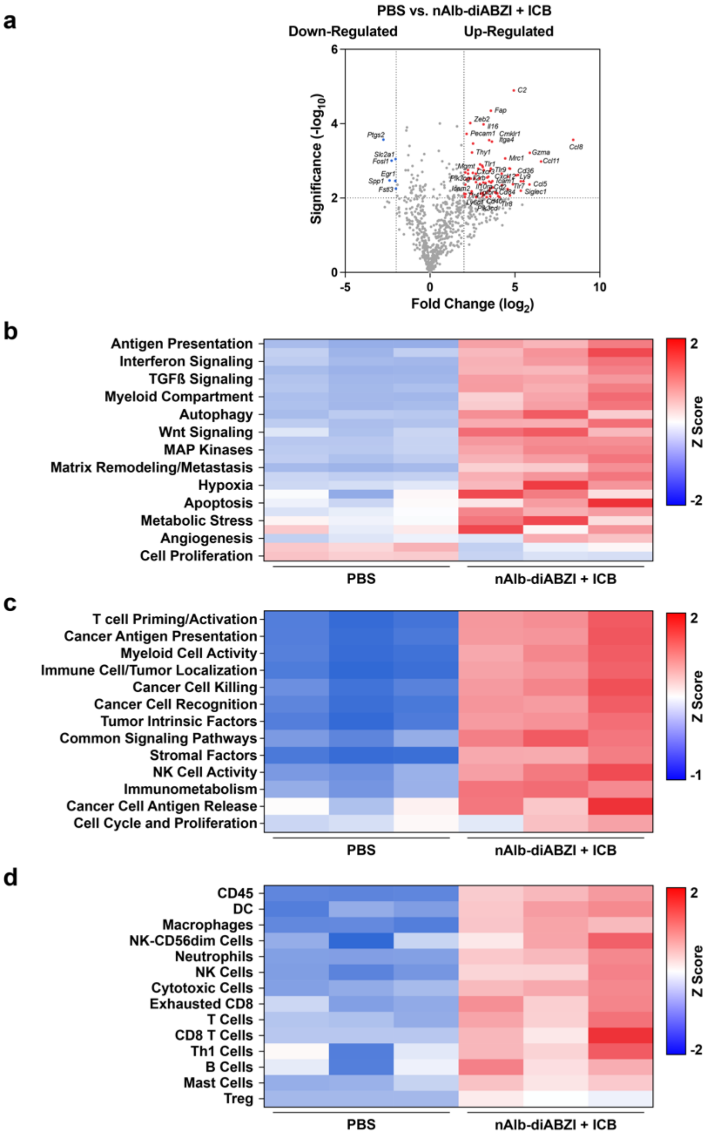

Supplementary Figure 27: (a) Volcano plots representing significance (-log$_{10}$) and fold change (log$_2$) for gene expression analysis in nAlb-diABZI + ICB vs. PBS (n=3) **(c-d)** Heat maps of NanoString gene cluster matrices showing Z score fold changes for **(b)** functional gene annotations, **(c)** biological signatures, and **(d)** cell types. Replicates are biological.

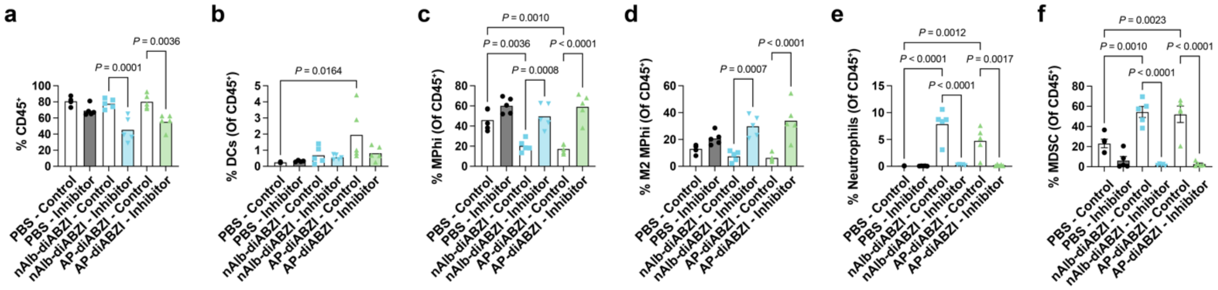

Supplementary Figure 28: Tumor flow cytometry analysis of EMT6 tumors following MDSC depletion using anti-GR-1 antibodies and treatment with PBS, nAlb-diABZI, and AP-diABZI by I.V. injection with 1.25 µg of STING agonist per dose. Frequency of **(a)** CD45+ cells, **(b)** CD11c+ DCs, **(c)** CD11b+F4/80+ macrophages, **(d)** CD11b+CD206+ M2-like macrophages, **(e)** neutrophils, and **(f)** MDSCs (n=5). *P* values were determined by one-way ANOVA with post-hoc Tukey's correction for multiple comparisons. Replicates are biological, and data are shown as mean ± SEM.

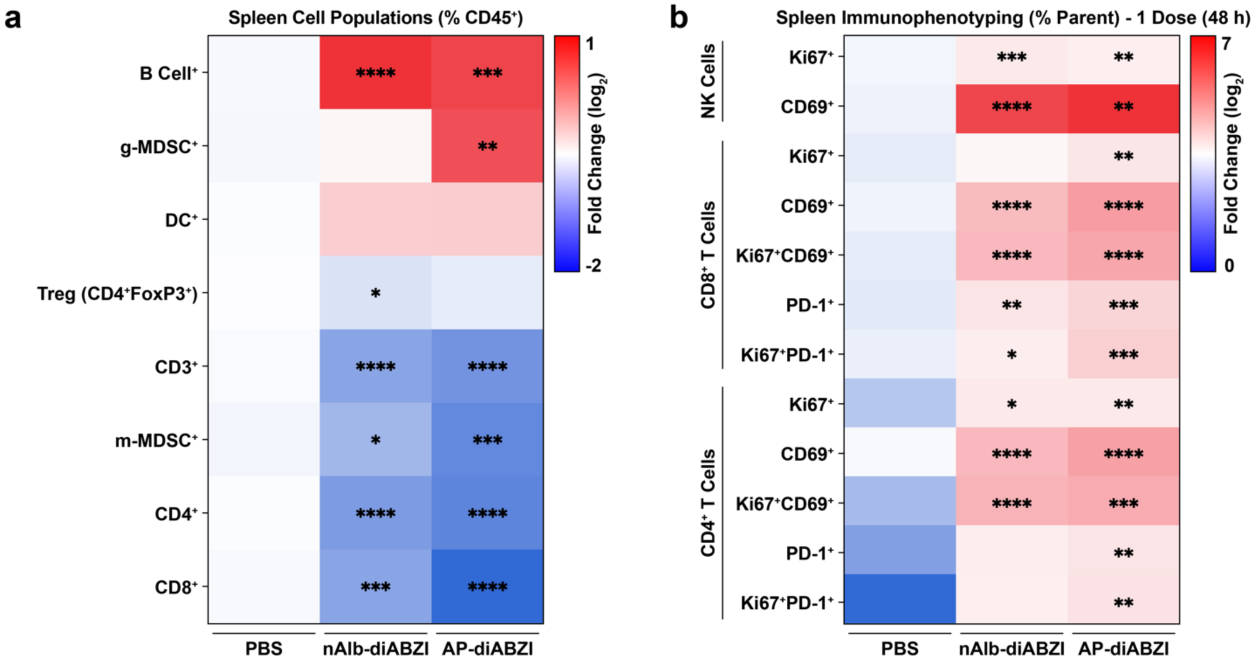

Supplementary Figure 29: Heat maps summarizing the fold change in the percentage of **(a)** indicated cell population and **(b)** frequency of NK cells, CD8$^+$ T cells, and CD4$^+$ T cells in the spleen expressing the indicated markers 48 h after treatment with one dose of nAlb-diABZI (n=8), AP-diABZI (n=8), or PBS (n=6) from EMT6 tumor-bearing Balb/c mice. *P* values were determined by one-way ANOVA with post-hoc Tukey's correction for multiple comparisons; *$P \leq 0.05$, **$P \leq 0.01$, ***$P \leq 0.001$, and ****$P < 0.0001$ compared to the PBS control. Replicates are biological.

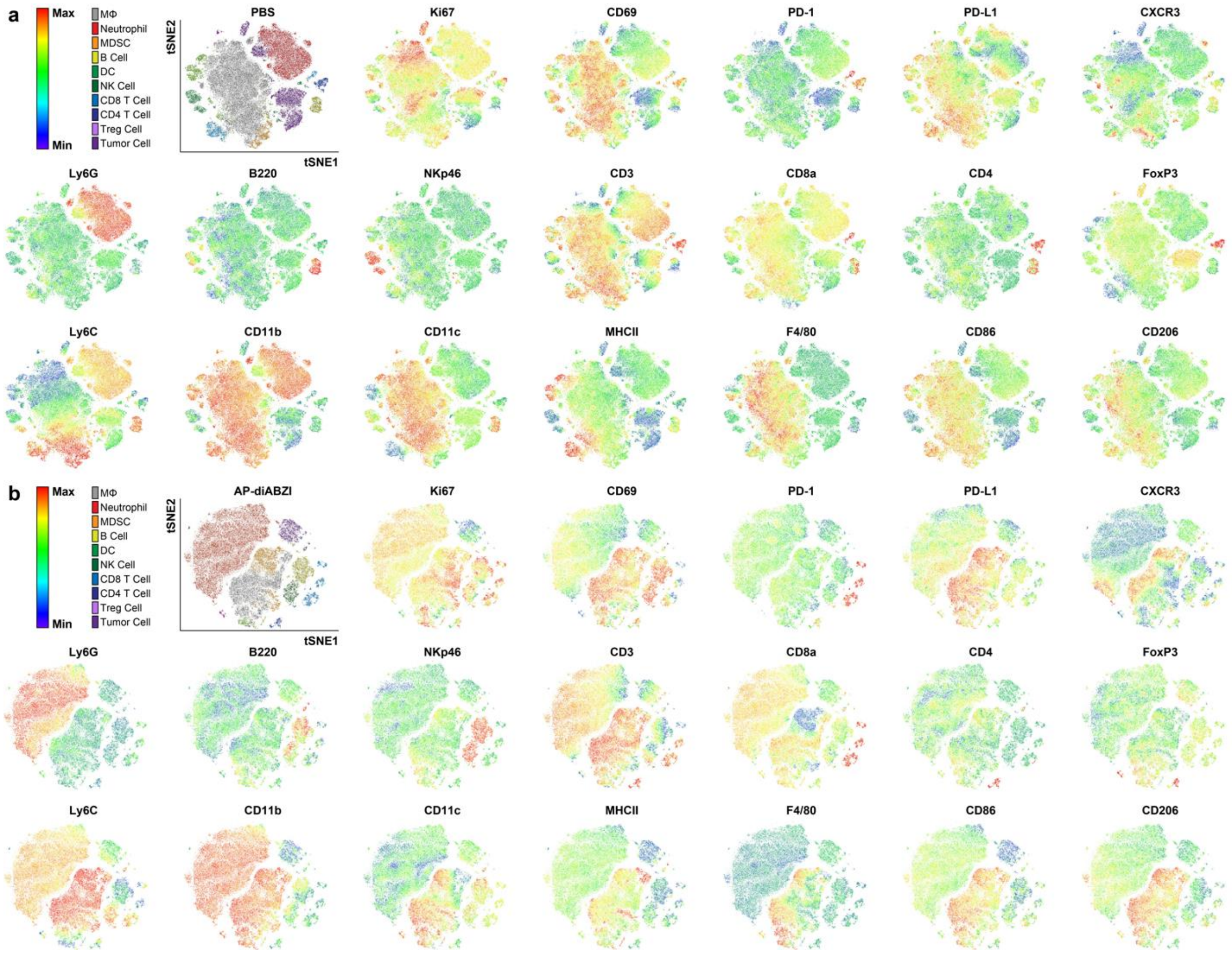

Supplementary Figure 30: tSNE plots of live cells from tumors of female Balb/c mice with EMT6 tumors after treatment with two doses of AP-diABZI (1.25 µg diABZI) or PBS. Maps are colored by indicated cell populations with relative expression levels. DC: dendritic cell; Mφ: macrophage; NK: natural killer cell; MDSC: myeloid-derived suppressor cells.

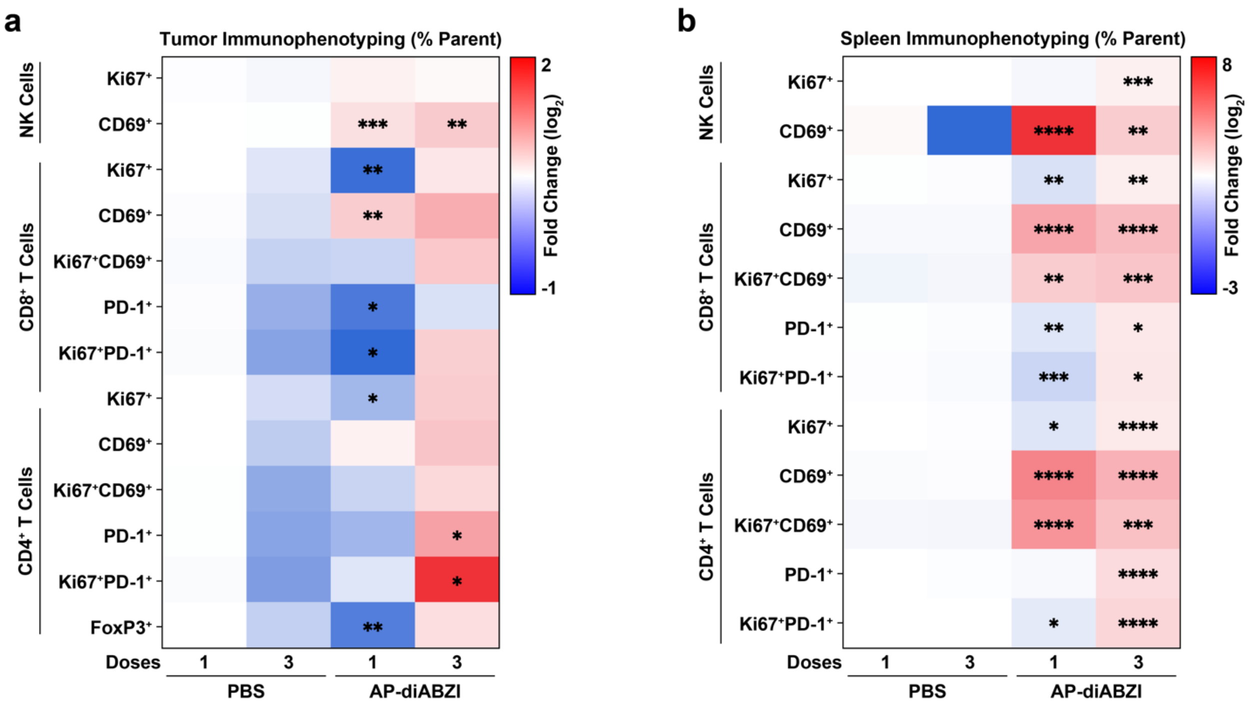

Supplementary Figure 31: Heat maps summarizing the fold change in the frequency of NK cells, $CD8^+$ T cells, and $CD4^+$ T cells expressing the indicated markers in **(a)** tumors and **(b)** spleens 24 h after treatment of Balb/c mice with EMT6 tumors with either one or three doses of nAlb-diABZI or PBS; n=7 for single dose PBS; n=8 for single dose AP-diABZI; n=6 for three dose PBS and AP-diABZI. *P* values determined by one-way ANOVA with post-hoc Tukey's correction for multiple comparisons; **P*≤0.05, ***P*≤0.01, ****P*≤0.001, and *****P*<0.0001 compared to the PBS control matched to the same number of doses. Replicates are biological.

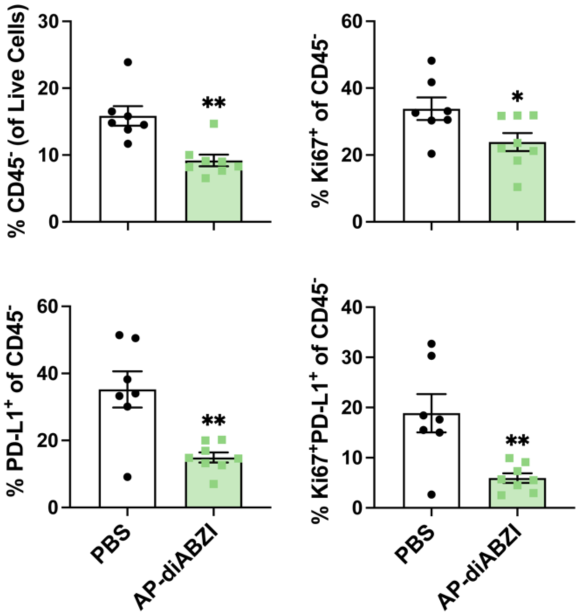

Supplementary Figure 32: Analysis of the frequency of live $CD45^-$ cells, and the frequency of PD-L1 and Ki67 expressing $CD45^-$ cells within EMT6 tumors after two doses of AP-diABZI (n=8) or PBS (n=7). **P*<0.05 and ***P*<0.01 indicates a statistically significant difference between PBS and AP-diABZI treated groups as determined by two-tailed Student's t-test. Replicates are biological, and data are shown as mean ± SEM.

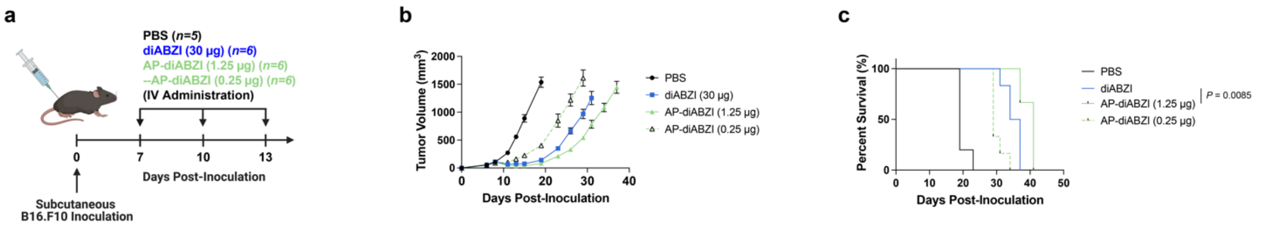

Supplementary Figure 33: (a) Schematic of B16.F10 tumor inoculation and treatment schedule for PBS, diABZI (30 µg) and two doses of AP-diABZI (1.25 µg and 0.25 µg). **(b)** Tumor growth curves and **(c)** Kaplan-Meier survival plots (n=5 for PBS; n=6 for all other groups). (c) Endpoint criteria of 1500 mm^3 tumor volume with *P* value determined by log-rank test for AP-diABZI (1.25 µg) compared to diABZI. Replicates are biological, and data are shown as mean ± SEM.

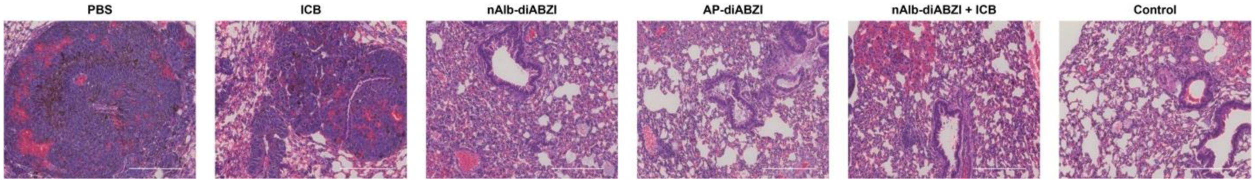

Supplementary Figure 34: Representative images of H&E-stained lung sections from mice with B16.F10 lung metastases treated as indicated or lungs from healthy mice (control), corresponding to the study presented in Figure 8a-e.

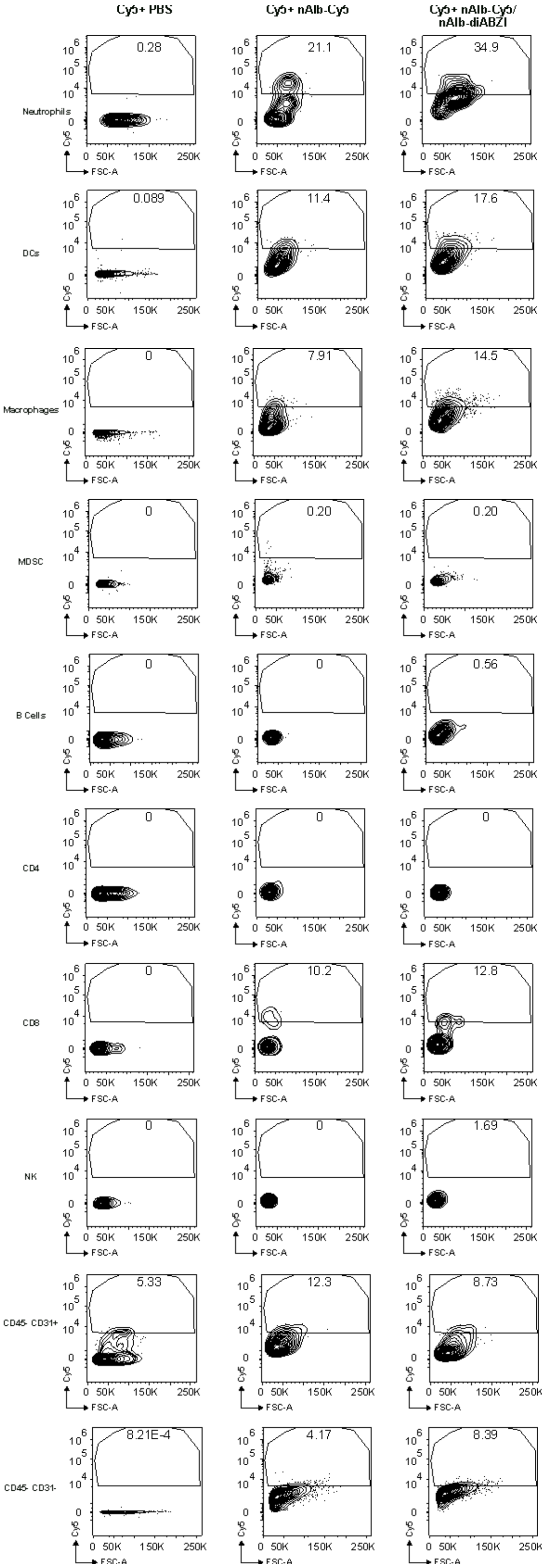

Supplementary Figure 35: Representative flow cytometry dot plots and gating strategy for **Fig. 2k,l** showing the relative uptake of Cy5 in each cell population in the tumor.

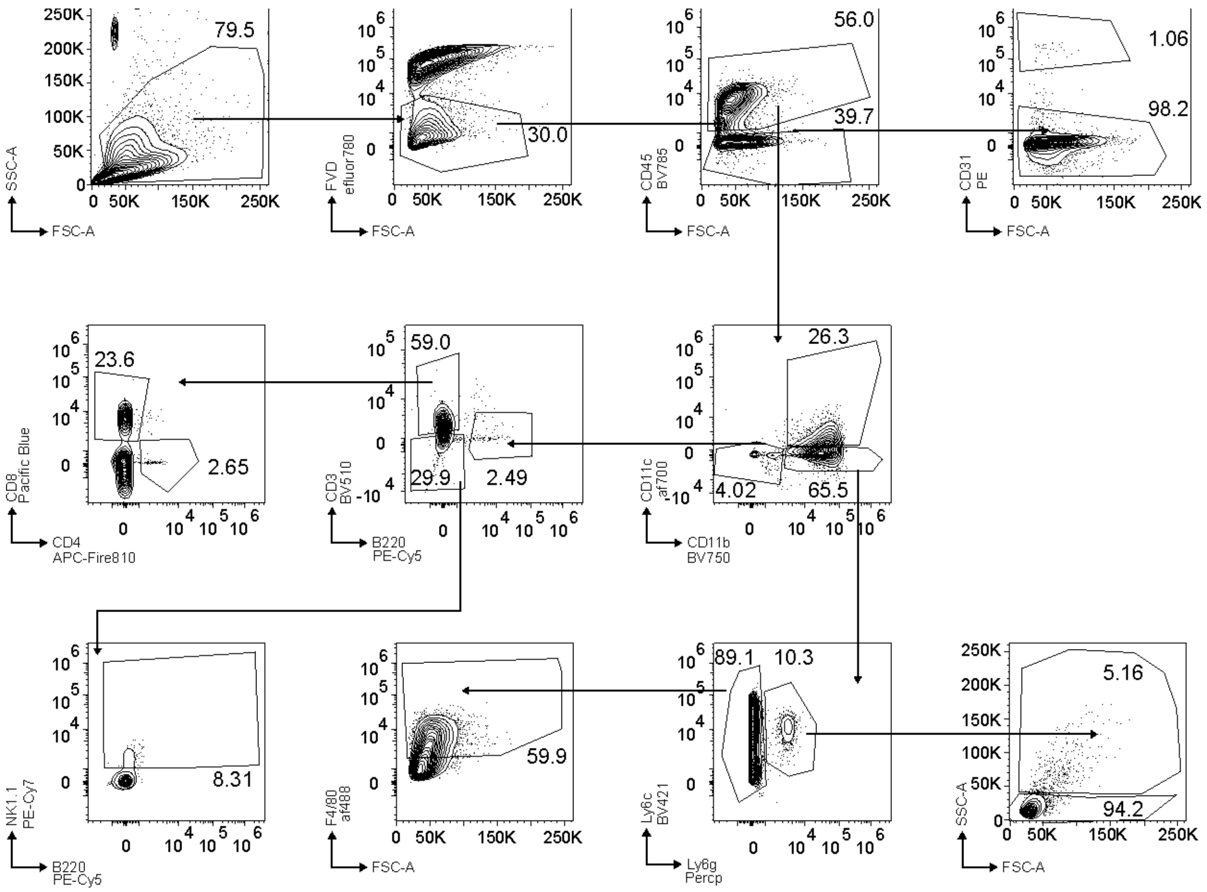

Supplementary Figure 36: Representative flow cytometry dot plots and gating strategy for **Fig. 2k,l** showing the cell populations present in the tumor.

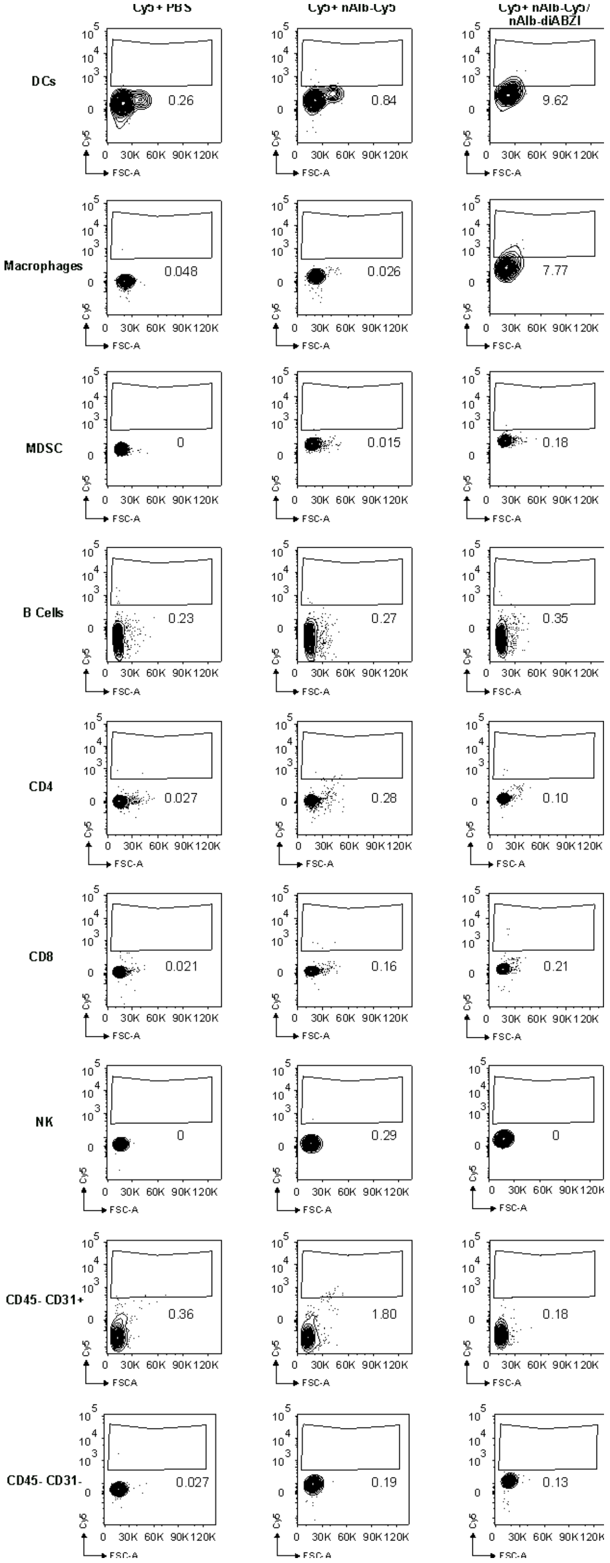

Supplementary Figure 37: Representative flow cytometry dot plots and gating strategy for **Fig. 2k,l** showing the relative uptake of Cy5 in each cell population in the spleen.

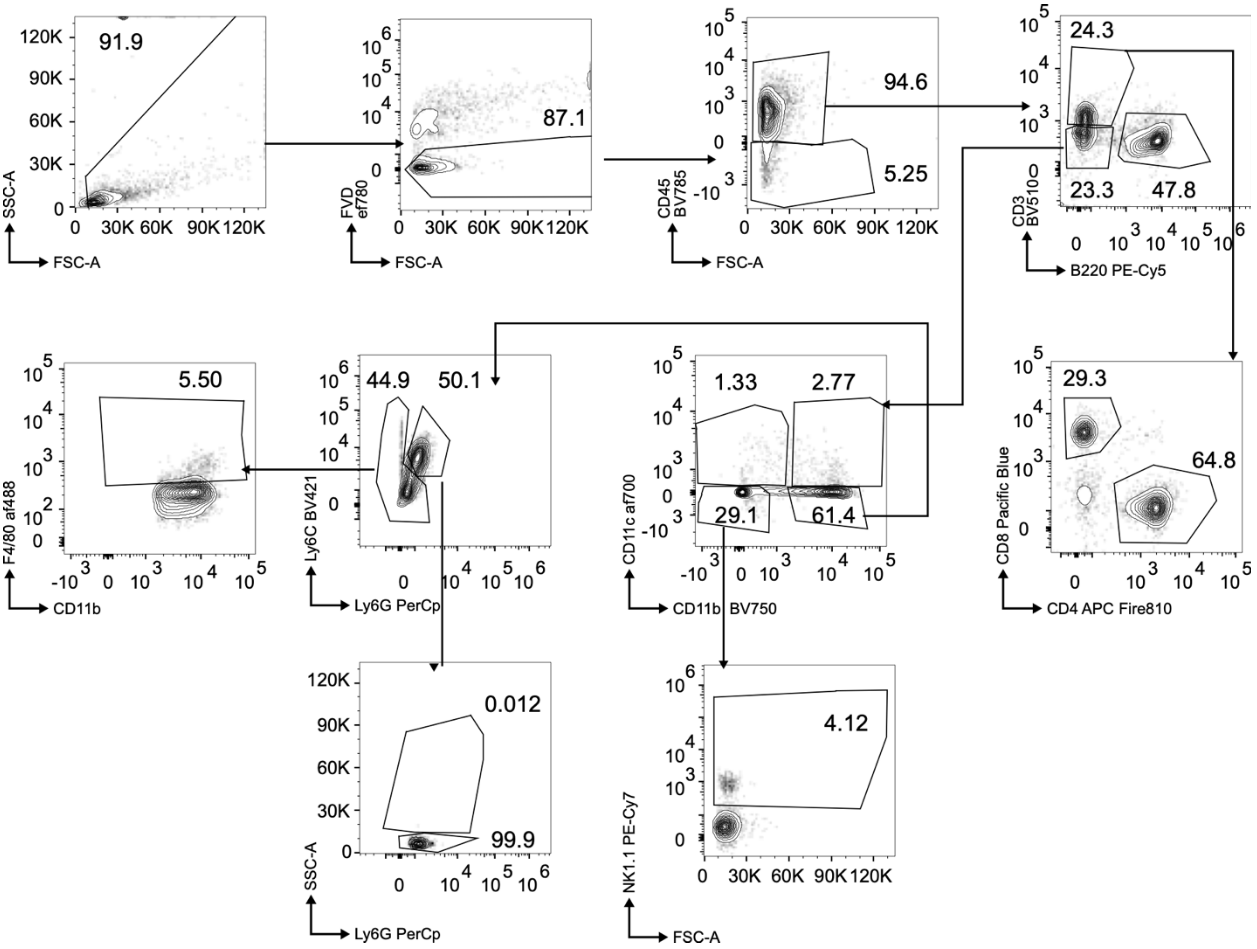

Supplementary Figure 38: Representative flow cytometry analysis for cell population mapping and immunophenotype determination within tumors and spleens from EMT6 bearing mice.

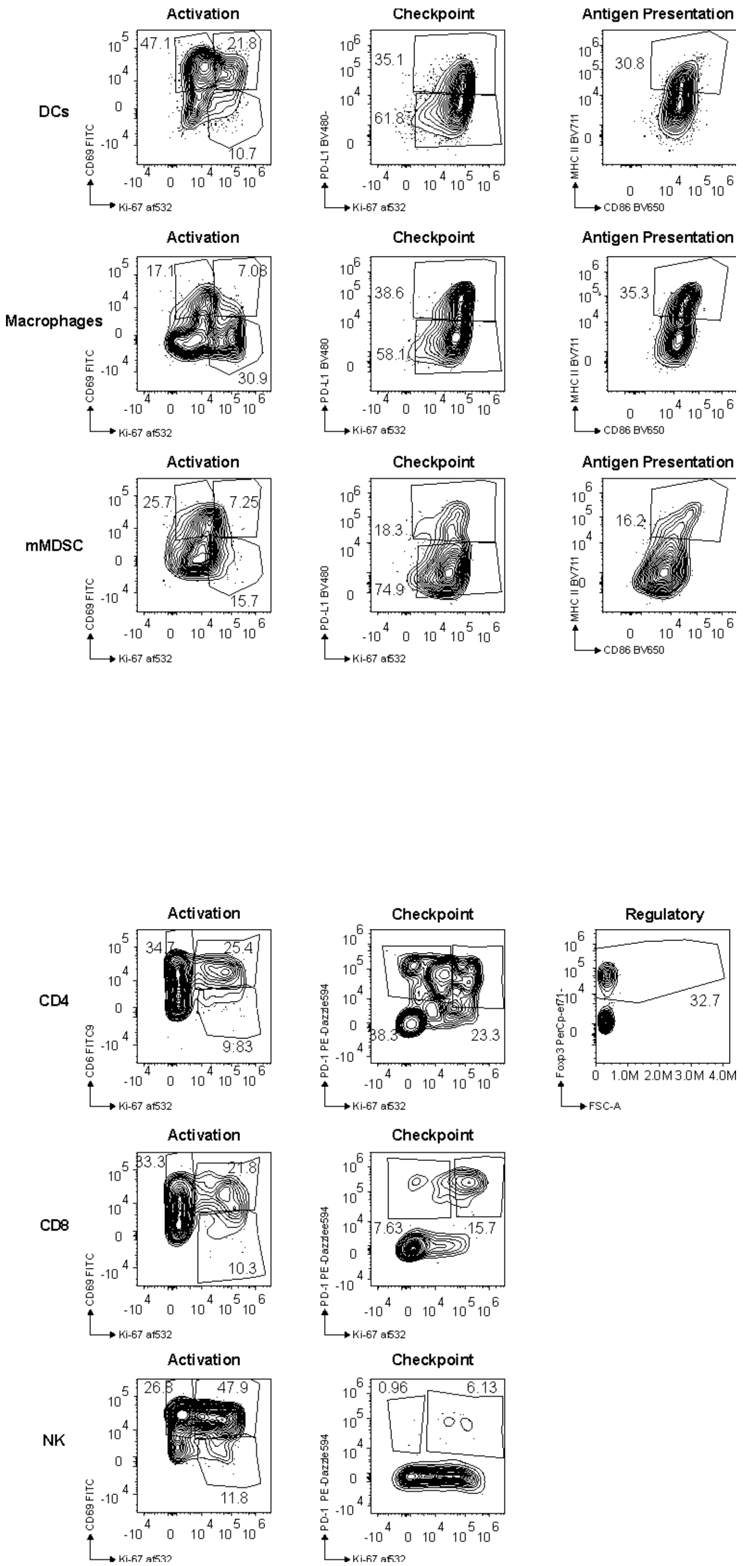

Supplementary Figure 39: Representative flow cytometry analysis denoting activation, proliferation, antigen presentation, and checkpoint markers for CD45+ cells, as broken down by cell population.

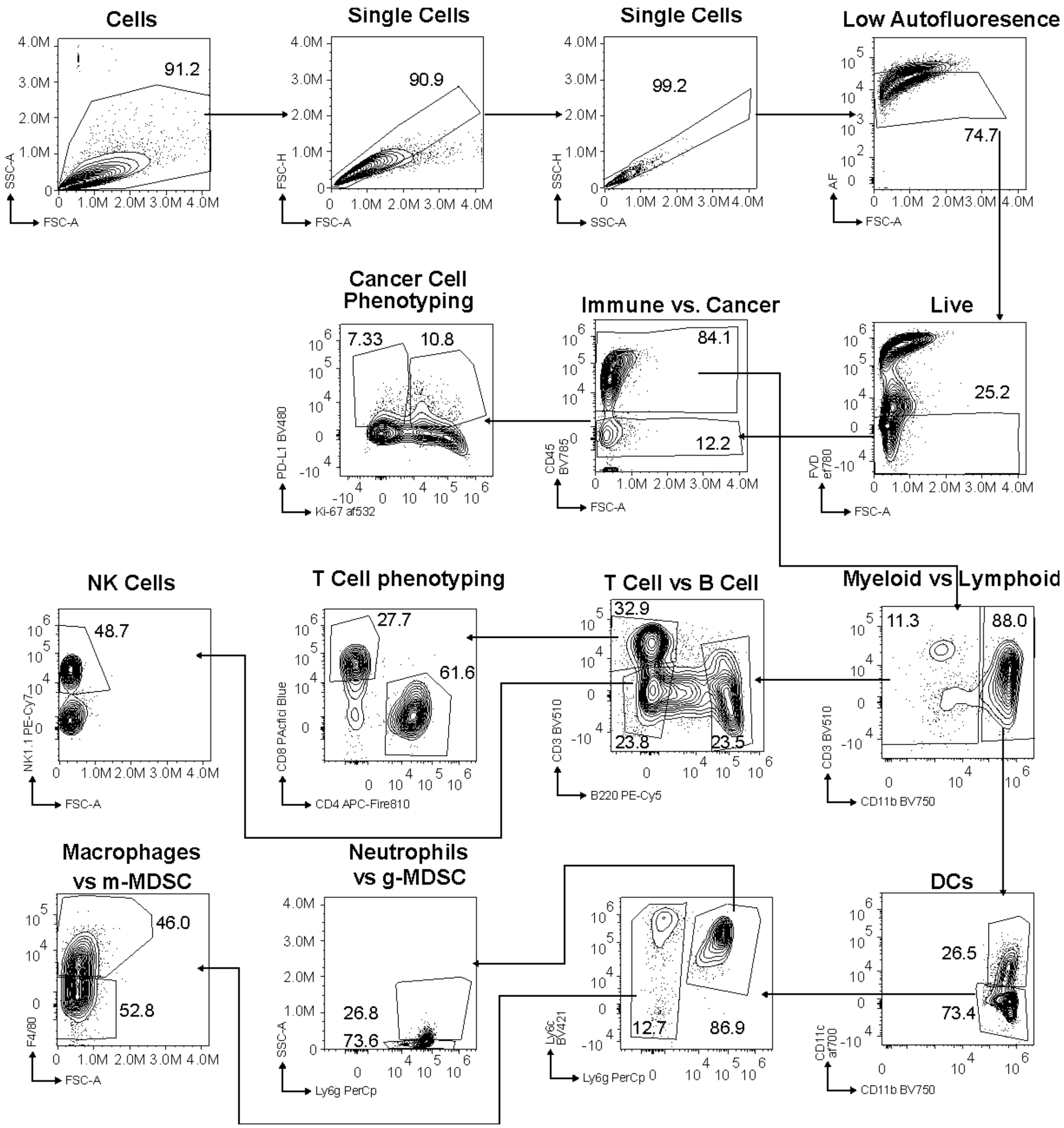

Supplementary Figure 40: Representative flow cytometry analysis to map cell populations for tumors and spleens from EMT6 bearing mice (Balb/c).

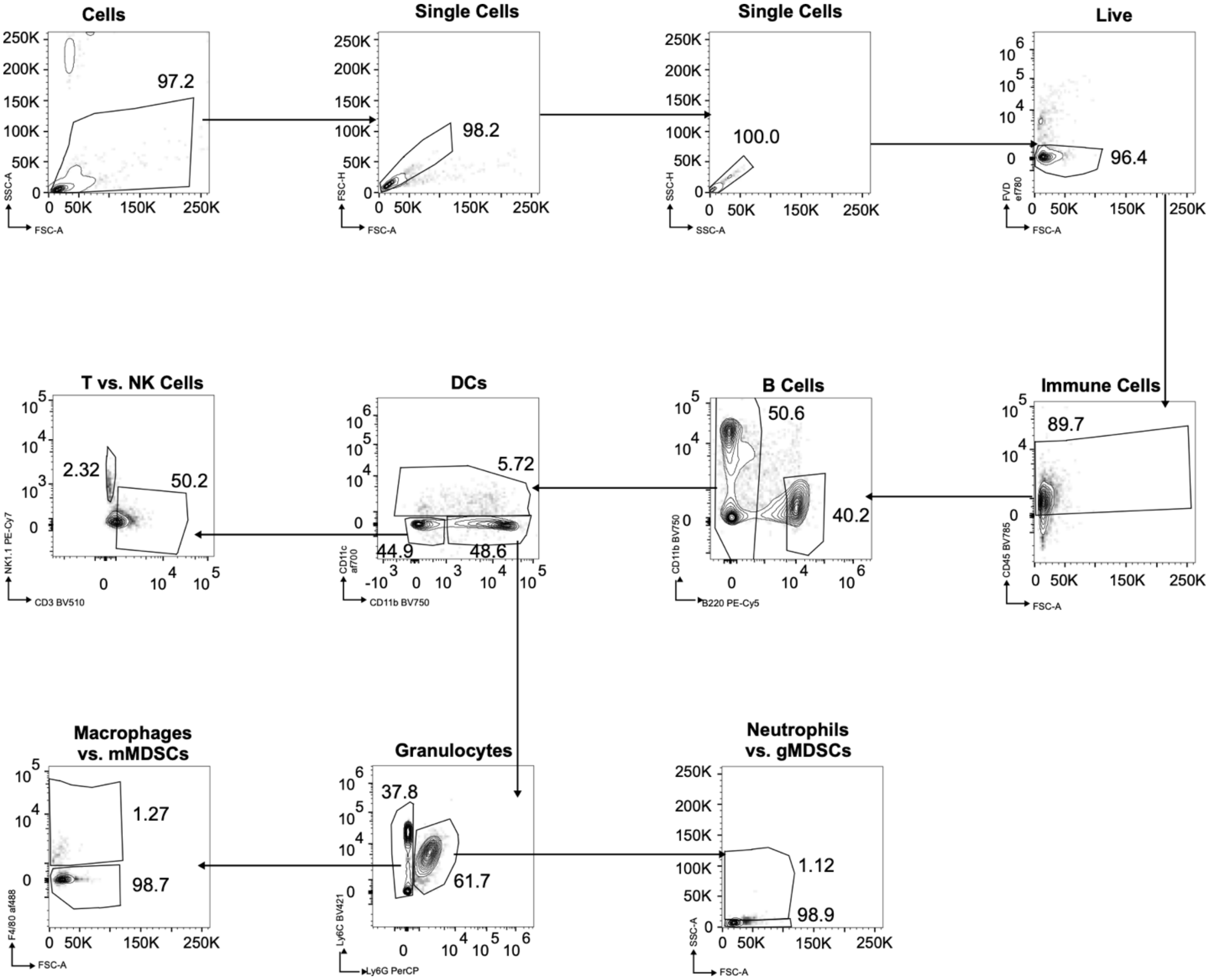

Supplementary Figure 41: Representative flow cytometric gating strategy for spleen cells from B16.F10 bearing mice (C57BL/6).

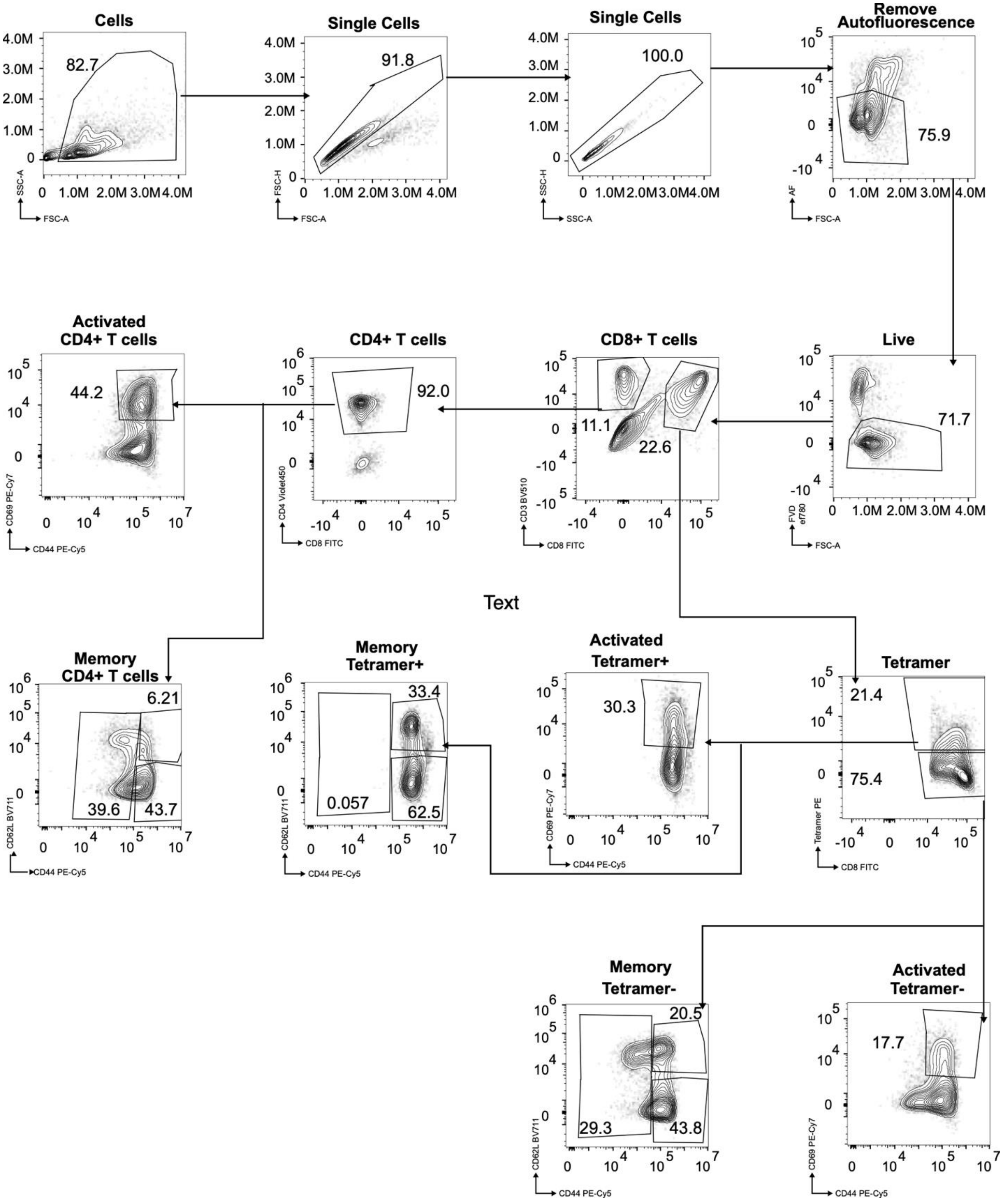

Supplementary Figure 42: Representative flow cytometric analysis of CD4, CD8 T cells, and SIINFEKL/H-2kB stained tetramer CD8 T cells, as well as both activation and memory T cell markers from B16.F10-OVA bearing C57BL/6 mice.

Supplementary Tables:

Supplementary Table 1: Protein sequences generated.

Protein Name	Sequence	Formula	Molecular Weight [Da]	Extinction Coefficient (ε_{280}) [M-1 cm-1]
**Engineered Sortase A (eSrtA) Sequence (No Start Codon)**<br><br>**eSrtA – HisTag**	**QAKPQIPKDKSKVAGYIEIPDADIKEPVYPGPATREQLNRGVSFAEENESLDDQNISIAGHTFIDRPNYQFTNLKAAKKGSMVYFKVGNETRKYKMTSIRNVKPTAVEVLDEQKGKDKQLTLITCDDYNEETGVWETRKIFVATEVKLEHHHHHH**	$C_{785}H_{1234}N_{220}O_{242}S_3$	17721.94	14440
**Anti-Albumin (nAlb) Sequence (No Start Codon)**<br><br>**nAlb – Ligation Tag**	**EVQLVESGGGLVQPGGSLRLSCAASGFTFRSFGMSWVRQAPGKEPEWVSSISGSGSDTLYADSVKGRFTISRDNAKTTLYLQMNSLKPEDTAVYYCTIGGSLSRSSQGTQVTVSSLPETGGHHHHHHEPEA**	$C_{606}H_{939}N_{175}O_{198}S_4$	13972.42	17085
**Anti-EGFR (nEGFR) Sequence (No Start Codon)**<br><br>**nEGFR – Ligation Tag**	**QVKLEESGGGSVQTGGSLRLTCAASGRTSRSYGMGWFRQAPGKEREFVSGISWRGDSTGYADSVKGRFTISRDNAKNTVDLQMNSLKPEDTAIYYCAAAAGSAWYGTLYEYDYWGQGTQVTVSSLPETGGHHHHHHEPEA**	$C_{662}H_{996}N_{194}O_{214}S_4$	15224.60	34045
**Anti-GFP (nGFP) Sequence (No Start Codon)**<br><br>**GFP – Ligation Tag**	**QVQLQESGGALVQPGGSLRLSCAASGFPVNRYSMRWYRQAPGKEREWVAGMSSAGDRSSYEDSVKGRFTISRDDARNTVYLQMNSLKPEDTAVYYCNVNVGFEYWGQGTQVTVSSLPETGGHHHHHHEPEA**	$C_{631}H_{955}N_{189}O_{200}S_5$	14548.97	27055
**Anti-PD-L1 (nPD-L1) Sequence (No Start Codon)**<br><br>**nPD-L1 – Ligation Tag**	**QVQLQESGGGLVHPGGSLRLSCATSGSIFSIISMGWYRQAPGKQRELVALVFRGGSTVYADSVKGRFTISGDIAKSTVYLQMDSLKPEDTAVYYCNAKPIGTAQYWGQGTQVTVSSLPETGGHHHHHHEPEA**	$C_{625}H_{966}N_{178}O_{192}S_4$	14173.86	20065
**Anti-Albumin – Anti-PD-L1 (AP) Sequence (No Start Codon)**<br><br>**nAlb – XTEN Linker – nPD-L1 – Ligation Tag**	**EVQLVESGGGLVQPGGSLRLSCAASGFTFRSFGMSWVRQAPGKEPEWVSSISGSGSDTLYADSVKGRFTISRDNAKTTLYLQMNSLKPEDTAVYYCTIGGSLSRSSQGTQVTVSSSGSETPGTSESAQVQLQESGGGLVHPGGSLRLSCATSGSIFSIISMGWYRQAPGKQRELVALVFRGGSTVYADSVKGRFTISGDIAKSTVYLQMDSLKPEDTAVYYCNAKPIGTAQYWGQGTQVTVSSLPETGGHHHHHHEPEA**	$C_{1195}H_{1863}N_{337}O_{388}S_8$	27415.44	37150

Supplementary Table 2: Antibodies for western blots.

Marker	Supplier	Catalog	Dilution Ratio
SPARC (D10F10) Rabbit mAb	Cell signaling	8725S	1:500
HSP90 (C45G5) Rabbit mAb	Cell signaling	4877S	1:2000
Anti-rabbit IgG, HRP-linked Antibody	Cell signaling	7074S	1:5000

Supplementary Table 3: Cy5 uptake flow cytometry panel (Balb/c).

Marker	Fluorochrome	Dilution	Company	Catalog
CD206	BV605	100	Biolegend	141721
CD3	BV510	40	Biolegend	100353
CD4	APC/Fire810	200	Biolegend	100480
CD8	Pacific blue	100	Biolegend	100725
B220	PE/CY5	200	Biolegend	103210
NKp46(BALB/C)	PE/CY7	100	Biolegend	137618
FOXP3	PerCP-eFluor™ 710	100	eBiosciences/Invitrogen	46-5773-82
CD69	FITC	100	Biolegend	104506
PD-1	PE-DAZZLE 594	100	Biolegend	135228
KI67	AF532	100	eBiosciences/Invitrogen	58-5698-82
CD11B	BV750	200	Biolegend	101267
CD11C	AF700	100	Biolegend	117320
LY6G	PERCP	100	Biolegend	127654
LY6C	BV421	200	Biolegend	128032
I-A/I-E(MHC II)	BV711	100	Biolegend	107643
F4/80	AF488	100	Biolegend	123120
CD31	PE	100	Biolegend	102507
CD45.2	BV785	100	Biolegend	109839
PD-L1	BV480	100	BD	748275
Viability	Eflour780	20000	Invitrogen	740614
CD86	BV650	100	Biolegend	105036

Supplementary Table 4: Cell population and immunophenotyping flow cytometry panel (Balb/c).

Marker	Fluorochrome	Dilution	Company	Catalog
CD206	BV605	100	Biolegend	141721
CD3	BV510	40	Biolegend	100353
CD4	APC/Fire810	200	Biolegend	100480
CD8	Pacific blue	100	Biolegend	100725
B220	PE/CY5	200	Biolegend	103210
NKp46	PE/CY7	100	Biolegend	137618
FOXP3	PerCP-eFluor™ 710	100	eBiosciences/Invitrogen	46-5773-82
CD69	FITC	100	Biolegend	104506
PD-1	PE-DAZZLE 594	100	Biolegend	135228
KI67	AF532	100	eBiosciences/Invitrogen	58-5698-82
CD11B	BV750	200	Biolegend	101267
CD11C	AF700	100	Biolegend	117320
LY6G	PERCP	100	Biolegend	127654
LY6C	BV421	200	Biolegend	128032
I-A/I-E(MHC II)	BV711	100	Biolegend	107643
F4/80	APC	100	Biolegend	123116
CXCR3 (CD183)	PE	100	Biolegend	126506
CD45.2	BV785	100	Biolegend	109839
PD-L1	BV480	100	BD	748275
Viability	Eflour780	20000	Invitrogen	740614
CD86	BV650	100	Biolegend	105036

Supplementary Table 5: Antibodies for immune cell memory in B16.F10-OVA tumor model.

**Marker**	**Fluorochrome**	**Dilution**	**Company**	**Catalog**
Viability	Eflour780	20000	eBiosciences/Invitrogen	65-0865-14
CD3	BV510	40	Biolegend	100234
CD8	FITC	100	Invitrogen	MA5-16759
CD4	violetFluor™ 450	100	Fisher Scientific	75-0042
CD69	PE/Cy7	100	Biolegend	104511
CD44	PE/CY7	100	Biolegend	103029
CD62L	BV711	100	Biolegend	104445
pOVA/H-2Kb Tetramer	PE	100	Prepared in-house	Sequence: $OVA_{257–264}$
