# Supplementary material for: Potentiating cancer immunotherapies with modular albumin-hitchhiking nanobody–STING agonist conjugates
Source: Nat Biomed Eng. 2025 Jun 11;9(10):1719–39. doi: 10.1038/s41551-025-01400-0 (PMC12532571; doi:10.1038/s41551-025-01400-0)
Supplement: Supplementary file 2 — Reporting Summary [file 41551_2025_1400_MOESM2_ESM.pdf]

nature portfolio

Corresponding author(s): John T. Wilson

Last updated by author(s): 09/23/2024

Reporting Summary

Nature Portfolio wishes to improve the reproducibility of the work that we publish. This form provides structure for consistency and transparency in reporting. For further information on Nature Portfolio policies, see our Editorial Policies and the Editorial Policy Checklist.

Please do not complete any field with "not applicable" or n/a. Refer to the help text for what text to use if an item is not relevant to your study.
For final submission: please carefully check your responses for accuracy; you will not be able to make changes later.

Statistics

For all statistical analyses, confirm that the following items are present in the figure legend, table legend, main text, or Methods section.

n/a	Confirmed	
[ ]	[X]	The exact sample size (*n*) for each experimental group/condition, given as a discrete number and unit of measurement
[ ]	[X]	A statement on whether measurements were taken from distinct samples or whether the same sample was measured repeatedly
[ ]	[X]	The statistical test(s) used AND whether they are one- or two-sided<br>*Only common tests should be described solely by name; describe more complex techniques in the Methods section.*
[X]	[ ]	A description of all covariates tested
[ ]	[X]	A description of any assumptions or corrections, such as tests of normality and adjustment for multiple comparisons
[ ]	[X]	A full description of the statistical parameters including central tendency (e.g. means) or other basic estimates (e.g. regression coefficient) AND variation (e.g. standard deviation) or associated estimates of uncertainty (e.g. confidence intervals)
[ ]	[X]	For null hypothesis testing, the test statistic (e.g. *F*, *t*, *r*) with confidence intervals, effect sizes, degrees of freedom and *P* value noted<br>*Give P values as exact values whenever suitable.*
[X]	[ ]	For Bayesian analysis, information on the choice of priors and Markov chain Monte Carlo settings
[X]	[ ]	For hierarchical and complex designs, identification of the appropriate level for tests and full reporting of outcomes
[X]	[ ]	Estimates of effect sizes (e.g. Cohen's *d*, Pearson's *r*), indicating how they were calculated

Our web collection on statistics for biologists contains articles on many of the points above.

Software and code

Policy information about availability of computer code

Data collection	ROSIE (Version 3), Agilent MassHunter IM-MS Acquisition Data software, NanoAnalyze TA Instruments Affinity ITC instrument, FlexControl (Version 4.0, BioTek SynergyHT Version 3.4, Bio-Rad CFX manager Software Version 3.0.
Data analysis	FlowJo Version 10, GraphPad PRISM Version 10, R (Version 4.4.1), PyMOL (Version 2.5.1), FlexAnalysis (Version 4.0), Mnova 14.1.1, FIJI Version 2.9.

For manuscripts utilizing custom algorithms or software that are central to the research but not yet described in published literature, software must be made available to editors and reviewers. We strongly encourage code deposition in a community repository (e.g. GitHub). See the Nature Portfolio guidelines for submitting code & software for further information.

Data

Policy information about availability of data

All manuscripts must include a data availability statement. This statement should provide the following information, where applicable:

- Accession codes, unique identifiers, or web links for publicly available datasets
- A description of any restrictions on data availability
- For clinical datasets or third party data, please ensure that the statement adheres to our policy

All materials used or generated in this study are available to researchers following appropriate standard material transfer agreements. Source Data are available within FigShare at the following DOI; 10.6084/m9.figshare.27098866. Modeled PDB structures used are 4N0F and 1AO6.

Research involving human participants, their data, or biological material

Policy information about studies with human participants or human data. See also policy information about sex, gender (identity/presentation), and sexual orientation and race, ethnicity and racism.

Reporting on sex and gender	No human participants or biological material was used.
Reporting on race, ethnicity, or other socially relevant groupings	No human participants or biological material was used.
Population characteristics	No human participants or biological material was used.
Recruitment	No human participants or biological material was used.
Ethics oversight	No human participants or biological material was used.

Note that full information on the approval of the study protocol must also be provided in the manuscript.

Field-specific reporting

Please select the one below that is the best fit for your research. If you are not sure, read the appropriate sections before making your selection.

[X] Life sciences [ ] Behavioural & social sciences [ ] Ecological, evolutionary & environmental sciences

For a reference copy of the document with all sections, see nature.com/documents/nr-reporting-summary-flat.pdf

Life sciences study design

All studies must disclose on these points even when the disclosure is negative.

Sample size	Sample sizes were determined by pilot experiments and through previous experiments in order to obtain statistically significant representative data.
Data exclusions	Data were only excluded by outlier analysis using GraphPad PRISM Version 10, as deemed appropriate. Mice were not excluded from studies.
Replication	All studies in the paper were repeated at least two times. No reported study failed upon repeat.
Randomization	All samples and organisms were randomly allocated into experimental groups.
Blinding	Blinding was not implemented formally in the study due to constraints in resources and personnel. The investigator responsible for organizing the experimental groups and handling sample collection was aware of the group allocations; however, colleagues who assisted with data collection were blinded to these details.

Behavioural & social sciences study design

All studies must disclose on these points even when the disclosure is negative.

Study description	
Research sample	
Sampling strategy	
Data collection	
Timing	
Data exclusions	
Non-participation	
Randomization	

Ecological, evolutionary & environmental sciences study design

All studies must disclose on these points even when the disclosure is negative.

Study description

Research sample

Sampling strategy

Data collection

Timing and spatial scale

Data exclusions

Reproducibility

Randomization

Blinding

Did the study involve field work? [ ] Yes [ ] No

Field work, collection and transport

Field conditions

Location

Access & import/export

Disturbance

Reporting for specific materials, systems and methods

We require information from authors about some types of materials, experimental systems and methods used in many studies. Here, indicate whether each material, system or method listed is relevant to your study. If you are not sure if a list item applies to your research, read the appropriate section before selecting a response.

Materials & experimental systems

n/a	Involved in the study	
[ ]	[X]	Antibodies
[ ]	[X]	Eukaryotic cell lines
[X]	[ ]	Palaeontology and archaeology
[ ]	[X]	Animals and other organisms
[X]	[ ]	Clinical data
[X]	[ ]	Dual use research of concern
[X]	[ ]	Plants

Methods

n/a	Involved in the study	
[X]	[ ]	ChIP-seq
[ ]	[X]	Flow cytometry
[X]	[ ]	MRI-based neuroimaging

Antibodies

Antibodies used

All antibodies used include anti-CD206 (catalog 141721, Biolegend), anti-CD3 (catalog 100353, Biolegend), anti-CD4 (catalog 100480, Biolegend), anti-CD8 (catalog 100725, Biolegend), anti-B220 (catalog 103210, Biolegend), anti-NKp46 (catalog 137618, Biolegend), anti-FOXP3 (catalog , Invitrogen), anti-CD69 (catalog 104506, Biolegend), anti-PD-1 (catalog 135228, Biolegend), anti-Ki67 (catalog 58-5698-82, Invitrogen), anti-CD11b (catalog 101267, Biolegend), anti-CD11c (catalog 117320, Biolegend), anti-LY6G (catalog 127654, Biolegend), anti-LY6C (catalog 117320, Biolegend), anti-MHC-II (catalog 107643, Biolegend), anti-F4/80 (catalog 123120, Biolegend), anti-CD31 (catalog 102507, Biolegend), anti-CD45.2 (catalog 109839, Biolegend), anti-PD-L1 (catalog 748275, BD), anti-CD86 (catalog 105036, Biolegend), anti-CD183 (catalog 126506, Biolegend), anti-CD44 (catalog 103029, Biolegend), anti-CD62L (catalog 104445, Biolegend), viability due (catalog 740614, Invitrogen), anti-SPARC (catalog 8725T, cell signaling), anti-HSP90 (catalog 4877T, cell signaling), anti-IgG secondary (catalog 31460, ThermoFisher Scientific).

Validation

For generated nanobodies, in house validation was performed using flow cytometry or isothermal calorimetry. All antibodies utilized in this study are commercially sourced and have been previously employed for the same applications reported here. Each supplier performs quality control to guarantee the reliability and reproducibility of their products. Detailed validation information and relevant literature citations for each antibody are available on the datasheets provided by the manufacturers

Eukaryotic cell lines

Policy information about cell lines and Sex and Gender in Research

Cell line source(s)	A549-Duals, THP-1 Duals, and RAW 264.7 cells were purchased from InvivoGen. EMT6, B16.F10, and B16.F10-LUC2 were purchased from ATCC. B16.F10-OVA were a gift from Dr. Amanda Lund, and originally purchased from Vitro Biotech. Primary cells were harvested in house.
Authentication	Cell lines were authenticated by testing binding of known receptors using flow cytometry with commercial antibodies.
Mycoplasma contamination	Cell lines were tested routinely for mycoplasma, all cells were negative for mycoplasma.
Commonly misidentified lines (See ICLAC register)	The cell lines used in this study are not found on ICLAC register Version 11.

Palaeontology and Archaeology

Specimen provenance	
Specimen deposition	
Dating methods	

☐ Tick this box to confirm that the raw and calibrated dates are available in the paper or in Supplementary Information.

Ethics oversight	

Note that full information on the approval of the study protocol must also be provided in the manuscript.

Animals and other research organisms

Policy information about studies involving animals; ARRIVE guidelines recommended for reporting animal research, and Sex and Gender in Research

Laboratory animals	Female C57BL/6J and Balb/c, and female FVB/N-Tg (MMTV-PyVT)634Mul, mice were acquired from Jackson labs (6-8 weeks old; 6–20-week-old mice were used for challenge experiments). CD45.1+/– OT-I female mice were purchased from Jackson labs (4-8 weeks old; C57BL/6-Tg(TcraTcrb)1100Mjb/J strain). Mice were kept on a 12 light/12 dark cycle and temperatures of 65-75 °F with 40-60% humidity.
Wild animals	No wild animals were used in the study.
Reporting on sex	Studies indicated the sex of mice used.
Field-collected samples	No field-collected samples were used in the study.
Ethics oversight	All experiments were performed in accordance with protocols approved by the Institutional Animal Care and Use Committee within Vanderbilt University. IACUC protocols M1800155 and M2300004.

Note that full information on the approval of the study protocol must also be provided in the manuscript.

Clinical data

Policy information about clinical studies

All manuscripts should comply with the ICMJE guidelines for publication of clinical research and a completed CONSORT checklist must be included with all submissions.

Clinical trial registration	
Study protocol	
Data collection	
Outcomes	

Dual use research of concern

Policy information about dual use research of concern

Hazards

Could the accidental, deliberate or reckless misuse of agents or technologies generated in the work, or the application of information presented in the manuscript, pose a threat to:

No | Yes

- [ ] [ ] Public health
- [ ] [ ] National security
- [ ] [ ] Crops and/or livestock
- [ ] [ ] Ecosystems
- [ ] [ ] Any other significant area

Experiments of concern

Does the work involve any of these experiments of concern:

No | Yes

- [ ] [ ] Demonstrate how to render a vaccine ineffective
- [ ] [ ] Confer resistance to therapeutically useful antibiotics or antiviral agents
- [ ] [ ] Enhance the virulence of a pathogen or render a nonpathogen virulent
- [ ] [ ] Increase transmissibility of a pathogen
- [ ] [ ] Alter the host range of a pathogen
- [ ] [ ] Enable evasion of diagnostic/detection modalities
- [ ] [ ] Enable the weaponization of a biological agent or toxin
- [ ] [ ] Any other potentially harmful combination of experiments and agents

Plants

Seed stocks	
Novel plant genotypes	
Authentication	

ChIP-seq

Data deposition

- [ ] Confirm that both raw and final processed data have been deposited in a public database such as GEO.
- [ ] Confirm that you have deposited or provided access to graph files (e.g. BED files) for the called peaks.

Data access links<br>*May remain private before publication.*	
Files in database submission	
Genome browser session<br>(e.g. UCSC)	

Methodology

Replicates	
Sequencing depth	
Antibodies	
Peak calling parameters	
Data quality	
Software	

Flow Cytometry

Plots

Confirm that:

[X] The axis labels state the marker and fluorochrome used (e.g. CD4-FITC).

[X] The axis scales are clearly visible. Include numbers along axes only for bottom left plot of group (a 'group' is an analysis of identical markers).

[X] All plots are contour plots with outliers or pseudocolor plots.

[X] A numerical value for number of cells or percentage (with statistics) is provided.

Methodology

Sample preparation: As described in methods, at brief: EMT6 tumor bearing Balb/c and B16.F10-OVA bearing C57BL/6 mice were euthanized either 24 h or 48 h after final treatment. Spleens and tumors were harvested, weighed, and placed onto ice. Tumors were digested in RPMI 1640 media containing a tumor dissociation kit. Tumors were dissociated and incubated for 30 min at 37 ºC for complete digestion. Tumors and spleens were mashed and separated into single cell suspensions and red blood cells were used twice using ACK lysis buffer. Cells were resuspended in flow buffer (2% FBS and 50μM dasatinib), counted, and stained with Fc-block (aCD16/32, 2.4G2, Tonbo) for 15min at 4 ºC, and then stained with the appropriate antibodies for 1hr at 4 ºC. Cells were then washed again with FACS buffer, fixed with 2% paraformaldehyde for 10 min, washed again with FACS buffer containing AccuCheck counting beads.

Instrument: Flow cytomrtry data were analyzed on a Cytek Aurora flow cytometer and on an Amnex Luminex CellStream flow cytometer.

Software: All flow cytometry data were analyzed using FlowJo software (version 10; Tree Star; https://www.flowjo.com/solutions/flowjo).

Cell population abundance: Cell population abundance is shown in gating strategies.

Gating strategy: Gating strategies are shown in supplemental figures.

[X] Tick this box to confirm that a figure exemplifying the gating strategy is provided in the Supplementary Information.

Magnetic resonance imaging

Experimental design

Design type:

Design specifications:

Behavioral performance measures:

Imaging type(s):

Field strength:

Sequence & imaging parameters:

Area of acquisition:

Diffusion MRI [ ] Used [ ] Not used

Preprocessing

Preprocessing software:

Normalization:

Normalization template:

Noise and artifact removal:

Volume censoring:

Statistical modeling & inference

Model type and settings:

Effect(s) tested:

Specify type of analysis: [ ] Whole brain [ ] ROI-based [ ] Both

Statistic type for inference

(See Eklund et al. 2016)

Correction

Models & analysis

n/a	Involved in the study
☐	☐ Functional and/or effective connectivity
☐	☐ Graph analysis
☐	☐ Multivariate modeling or predictive analysis

Functional and/or effective connectivity

Graph analysis

Multivariate modeling and predictive analysis

This checklist template is licensed under a Creative Commons Attribution 4.0 International License, which permits use, sharing, adaptation, distribution and reproduction in any medium or format, as long as you give appropriate credit to the original author(s) and the source, provide a link to the Creative Commons license, and indicate if changes were made. The images or other third party material in this article are included in the article's Creative Commons license, unless indicated otherwise in a credit line to the material. If material is not included in the article's Creative Commons license and your intended use is not permitted by statutory regulation or exceeds the permitted use, you will need to obtain permission directly from the copyright holder. To view a copy of this license, visit http://creativecommons.org/licenses/by/4.0/
